# C5a impairs phagosomal maturation in the neutrophil through phosphoproteomic remodeling

**DOI:** 10.1172/jci.insight.137029

**Published:** 2020-08-06

**Authors:** Alexander J.T. Wood, Arlette M. Vassallo, Marie-Hélène Ruchaud-Sparagano, Jonathan Scott, Carmelo Zinnato, Carmen Gonzalez-Tejedo, Kamal Kishore, Clive S. D’Santos, A. John Simpson, David K. Menon, Charlotte Summers, Edwin R. Chilvers, Klaus Okkenhaug, Andrew Conway Morris

**Affiliations:** 1 Department of Medicine, University of Cambridge, Addenbrooke’s Hospital, Hills Road, Cambridge, United Kingdom.; 2 Faculty of Medical Sciences, Newcastle University, Framlington Place, Newcastle upon Tyne, United Kingdom.; 3 Cancer Research UK Cambridge Institute, University of Cambridge, Li Ka Shing Centre, Robinson Way, Cambridge, United Kingdom.; 4 Newcastle upon Tyne Hospitals NHS Foundation Trust, Queen Victoria Road, Newcastle upon Tyne, United Kingdom.; 5 National Heart and Lung Institute, Imperial College, London, United Kingdom.; 6 Division of Immunology, Department of Pathology, University of Cambridge, Tennis Court Road, Cambridge, United Kingdom.

**Keywords:** Immunology, Infectious disease, Bacterial infections, Complement, Neutrophils

## Abstract

Critical illness is accompanied by the release of large amounts of the anaphylotoxin, C5a. C5a suppresses antimicrobial functions of neutrophils which is associated with adverse outcomes. The signaling pathways that mediate C5a-induced neutrophil dysfunction are incompletely understood. Healthy donor neutrophils exposed to purified C5a demonstrated a prolonged defect (7 hours) in phagocytosis of *Staphylococcus aureus*. Phosphoproteomic profiling of 2712 phosphoproteins identified persistent C5a signaling and selective impairment of phagosomal protein phosphorylation on exposure to *S*. *aureus*. Notable proteins included early endosomal marker ZFYVE16 and V-ATPase proton channel component ATPV1G1. An assay of phagosomal acidification demonstrated C5a-induced impairment of phagosomal acidification, which was recapitulated in neutrophils from critically ill patients. Examination of the C5a-impaired protein phosphorylation indicated a role for the PI3K VPS34 in phagosomal maturation. Inhibition of VPS34 impaired neutrophil phagosomal acidification and killing of *S*. *aureus*. This study provides a phosphoproteomic assessment of human neutrophil signaling in response to *S*. *aureus* and its disruption by C5a, identifying a defect in phagosomal maturation and mechanisms of immune failure in critical illness.

## Abstract 2

C5a disrupts the neutrophil phosphoproteomic response to bacteria, impairing phagosomal maturation and bacterial killing.

## Graphical abstract

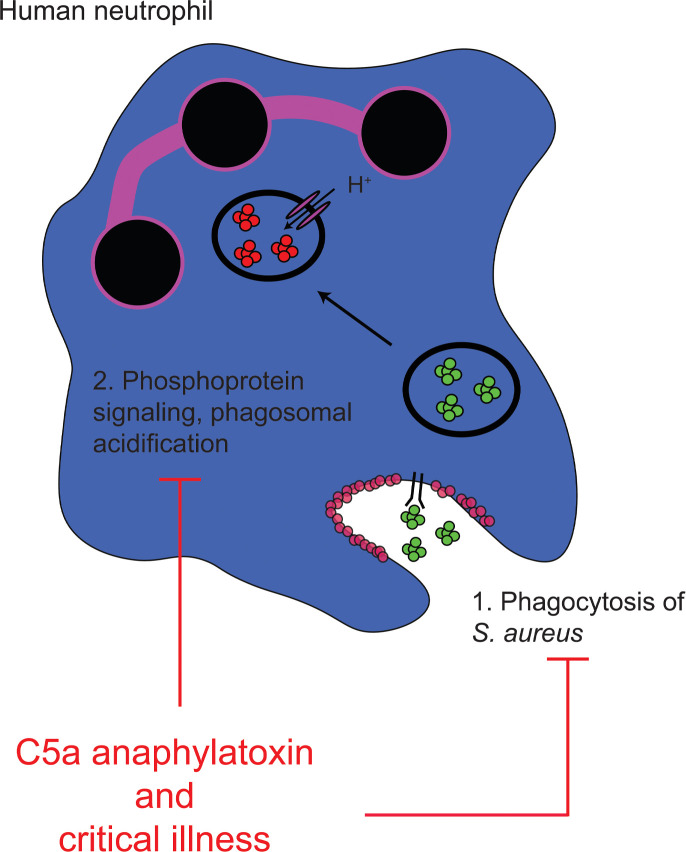

## Introduction

Critically ill patients who require exogenous organ support as a result of severe physiologic insult are at high risk of secondary infections (1). Critical illness may arise from a variety of sterile or infectious insults. However, despite its varied etiology, critical illness is often accompanied by stereotyped immune dysregulation, with features of both hyperinflammation and immune-mediated organ damage, as well as impairment of antimicrobial functions (2–4). Critical illness is estimated to cause 58 million adult deaths per year globally (5), and although much of the mortality is attributable to the underlying condition, secondary infections make a significant contribution to the eventual outcome (5–8).

Impairment of immune cell function predicts secondary infection (2, 9–11), and the failure of neutrophil phagocytosis and bacterial killing has been demonstrated to be one of the strongest predictors of these infections. A key driver of the functional impairment of neutrophils is the anaphylatoxin C5a (12–14). However, there remain no efficacious treatments for critical illness–induced immune dysfunction in part because the mechanisms that underpin C5a-induced dysfunction are incompletely understood.

A wealth of data has demonstrated the importance of C5a in driving classical inflammatory events in neutrophils, including chemotaxis (15, 16), generation of ROS (14, 17, 18), phagocytosis (19, 20), degranulation (21, 22), and delayed apoptosis (23–25). One of the key questions in this area is how to explain the divergent findings of C5a being not only a critical cofactor in phagocytosis (19, 20) but also capable of suppression of phagocytosis in sepsis and critical illness (12–14). In critical illness, dysregulated activation of the complement and coagulation cascades occurs, leading to exposure of neutrophils to high concentrations of C5a (4, 26–29). In these circumstances, we and others have shown that C5a reduces neutrophil phagocytosis and ROS production in both rodent models and critically ill patients (12–14, 30). Further, C5a exposure has been shown to be associated with nosocomial infection, organ failure, and increased mortality in critically ill patients (2, 12, 13, 30–32).

Although several signal-mediating aspects of C5a-induced neutrophil dysfunction have been established (13, 14, 21), a global picture of signaling in neutrophils encountering common pathogens and how this process is perturbed by C5a does not exist. Such studies are challenging in neutrophils owing to their high degradative enzyme content and short in vitro survival times (33).

This study aimed to characterize the neutrophil phosphoprotein response to a common nosocomial pathogen, *Staphylococcus aureus*, and investigate how this is perturbed by prior exposure to C5a. Our differential phosphoprotein analysis implicated C5a in altered phagosomal maturation, findings that we confirmed with functional neutrophil assays in C5a-treated healthy donor cells and those from critically ill patients. The phosphoprotein response to *S*. *aureus* implicated the involvement of the PI3K VPS34, and hence we continued examined the effects of this enzyme on phagosomal maturation.

## Results

### C5a induces a prolonged defect in neutrophil phagocytosis of bacteria.

**Figure 1:**
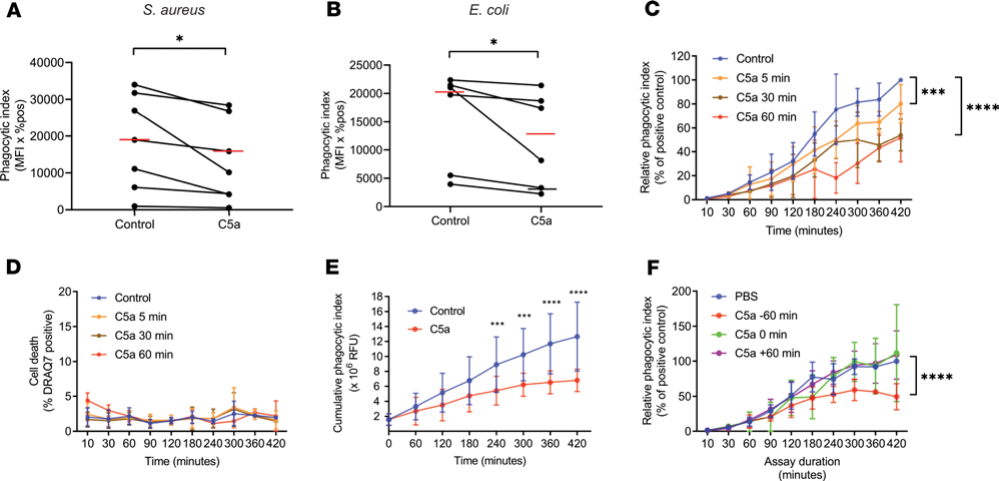
C5a induces a prolonged defect in neutrophil phagocytosis of bacteria. (**A** and **B**) Isolated neutrophils were pretreated with 100 nM C5a or vehicle control for 60 minutes before incubation with *S*. *aureus* (**A**) or *E*. *coli* (**B**) bioparticles (5 μg/mL). Data are presented as the median phagocytic index for each condition for *n* = 7 (**A**) or 6 (**B**) independent experiments. *P = 0.016 (**A**) and 0.031 (**B**) by Wilcoxon’s matched-pairs signed-rank test. (**C**) Neutrophils were pulsed with C5a (100 nM) or PBS control for the indicated periods of time and then washed 2 times. *S*. *aureus* bioparticles (5 μg/mL) were then added and cells were incubated for the indicated time points. Data are presented as the mean plus or minus SD of the phagocytic index of C5a-treated cells relative to their paired vehicle control for *n* = 5 independent experiments. *P* < 0.0001 for time and *P* = 0.0186 for treatment by 2-way ANOVA. ***P = 0.0001 ****P < 0.0001 by Dunnett’s multiple comparison test. (**D**) Data are presented as the mean plus or minus SD of the percentage of DRAQ7 positive, dead cells for *n* = 5 independent experiments. *P* = 0.378 for time and *P* = 0.349 for treatment by 2-way ANOVA. (**E**) Anticoagulated whole blood was pretreated with 300 nM C5a or control for the indicated duration before phagocytosis was measured as previously indicated. Data are presented as the mean plus or minus SD of the cumulative phagocytic index for *n* = 4 independent experiments. *P* < 0.0001 by 2-way ANOVA, ****P < 0.0001, ***P < 0.001 by Holm-Šidák’s multiple comparisons test. (**F**) *S*. *aureus* bioparticles (15 μg/mL) were incubated with isolated PMNs in the presence of C5a (100 nM) or PBS added at the indicated time points, with time 0 representing the time of addition of *S*. *aureus* bioparticles. Experiments proceeded for the indicated time points and phagocytic index quantified. Data are presented as the mean plus or minus SD of the phagocytic index of C5a-treated cells relative to their paired vehicle control for *n* = 5 independent experiments. *P* < 0.0001 for time and *P* = 0.0186 for treatment by 2-way ANOVA. ****P < 0.0001 by Dunnett’s multiple comparisons test.

C5a induces a defect in phagocytosis of the clinically relevant bacterial species *S*. *aureus* (Figure 1A) and *E*. *coli* (Figure 1B). Pulse exposure of neutrophils to C5a revealed a persistent defect in phagocytosis lasting at least 7 hours (Figure 1C), with short pulses inducing a significant defect. These effects were not explained by the loss of cell viability (Figure 1D). A similar prolonged defect was identified in the whole blood assay (Figure 1E), representing continuous exposure of neutrophils to C5a (which cannot be washed off in this assay). The ability of C5a to inhibit phagocytosis was dependent on the temporal relationship between C5a and *S*. *aureus* exposure. Only preexposure to C5a induced the defect in phagocytosis, whereas coexposure or the addition of C5a 30 minutes after *S*. *aureus* addition failed to induce a defect (Figure 1F). In a dose titration, we could not identify any dose that enhanced phagocytosis; instead we found progressive decrease in phagocytosis as concentration of C5a was increased (Supplemental Figure 1A; supplemental material available online with this article; https://doi.org/10.1172/jci.insight.137029DS1).

To explore the potential mechanisms whereby preexposure to *S*. *aureus* prevents the inhibitory effect of C5a, we examined whether this could be due to reduced C5aR1 expression. Although we could demonstrate a reduction in C5aR1 after *S*. *aureus* exposure (Supplemental Figure 1B), this was modest and similar to the reductions induced by other inflammatory mediators including LPS and leukotriene A (LTA), neither of which ameliorated the subsequent suppressive effect of C5a (Supplemental Figure 1C). Further, C5a — and not LPS, LTA, GM-CSF, and TNF — reduced neutrophil phagocytosis (Supplemental Figure 1D). To confirm the functional relevance of C5a-impaired phagocytosis, we demonstrated that C5a pretreatment reduced bacterial killing of *S*. *aureus* (Supplemental Figure 1E).

Given the temporal dependence of the inhibitory effect of C5a, we examined whether prior exposure to *S*. *aureus* enhanced or impaired subsequent ingestion. Using sequential exposure of whole blood to *S*. *aureus* labeled with 2 different pHrodo probes, we demonstrated that neutrophils that ingest the first target are more likely to ingest the second target (Supplemental Figure 2A), whereas progressive increase in the first target revealed a fundamental limit on the capacity of neutrophils to ingest (Supplemental Figure 2, B and C).

### S. aureus and C5a induce widespread changes in the neutrophil phosphoproteome.

Although key signaling “nodes” have been identified in neutrophils after C5a exposure (12, 13), no map of global signaling networks has been produced. Given the rapidity of the C5a-induced phagocytic impairment demonstrated above, and the known signaling kinetics of GPCRs (34), we examined posttranslational modification by phosphorylation (i.e., a phosphoproteomic approach).

**Figure 2:**
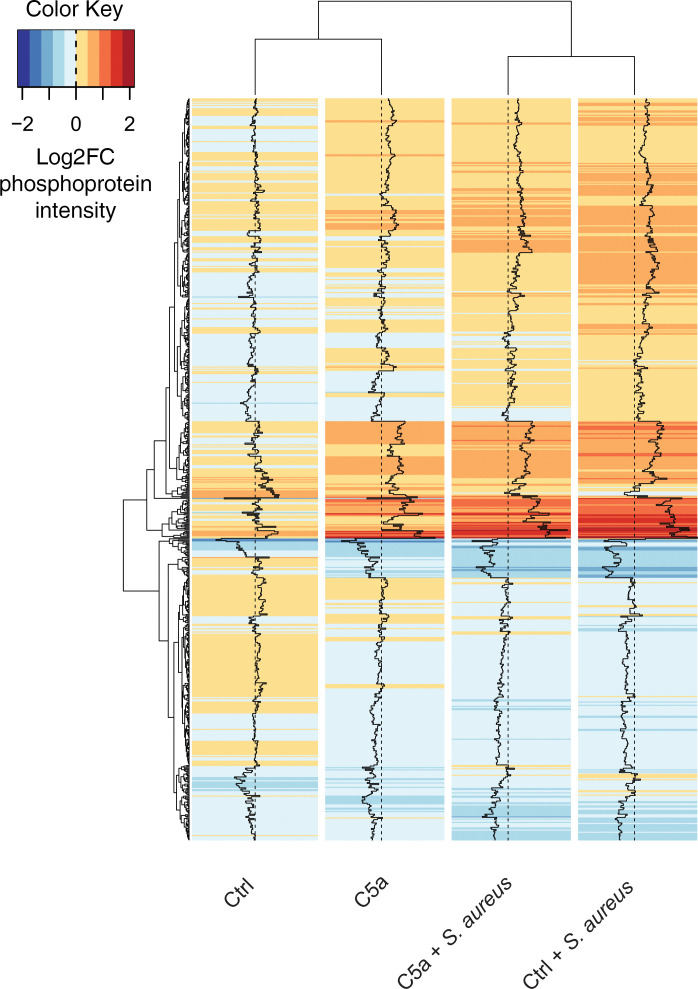
*S*. *aureus* and C5a induce widespread changes in the neutrophil phosphoproteome. Neutrophils were isolated from *n* = 4 healthy donors, pretreated with C5a (100 nM) or control before addition of pHrodo *S*. *aureus* bioparticles (15 μg/mL). Cells were allowed to phagocytose for 15 minutes before being snap frozen and processed for proteomic analysis as detailed in Methods. A heatmap of phosphoprotein intensity relative to baseline (log2FC) across the 4 experimental conditions shows phosphoproteins with variance across conditions in the top 75th percentile with dendrograms clustered by Euclidean distance. Increased phosphoprotein expression is indicated in red, decreased in blue. Only phosphoproteins detected in all 4 donor samples were included.

In total, 4859 proteins and 2712 phosphoproteins were identified in peripheral blood neutrophils obtained from 4 healthy volunteers. C5a-induced suppression of phagocytosis in these donors was confirmed (Supplemental Figure 3A), and technical reproducibility was high (Supplemental Figure 3, B–E) with the magnitude of phosphorylation changes within the previously reported range (35). Changes in the human proteome were minimal (2% of total proteome with *S*. *aureus* treatment), whereas phosphoprotein expression varied markedly (31.6% of total phosphoproteome with *S*. *aureus* treatment, Supplemental Table 1). Figure 2 shows the top 25% most variable phosphoproteins, which demonstrates the significant effect these stimuli have on neutrophil signaling. The phosphoproteomic and proteomic data sets are publicly available in the PRIDE database (https://www.ebi.ac.uk/pride/ reference PXD017092).

### C5a exposure induces persistent alteration in phosphoproteins across several pathways.

**Figure 3:**
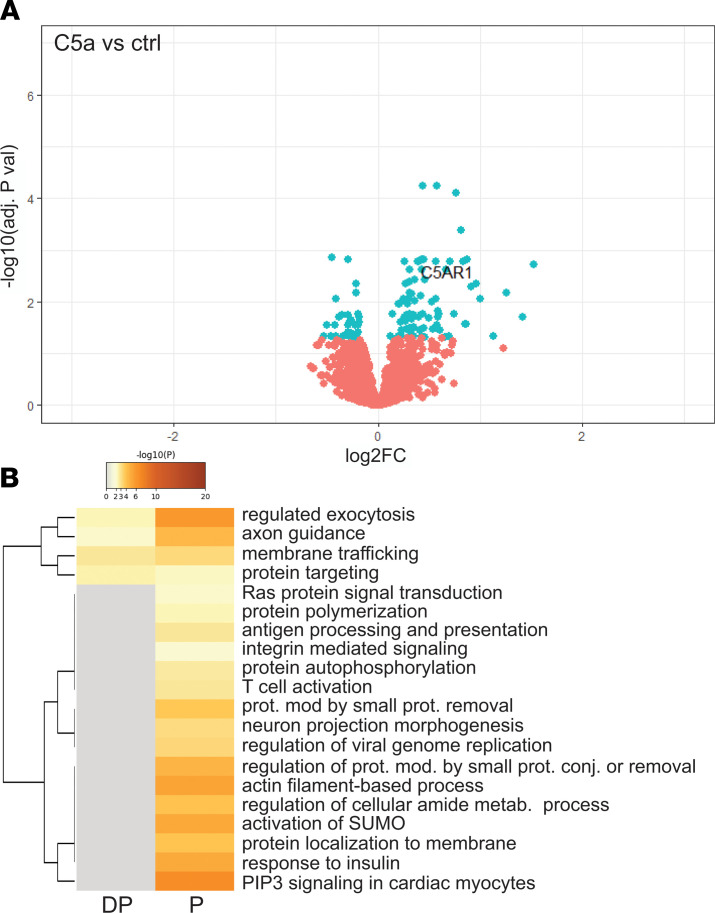
C5a exposure induces persistent alteration in phosphoproteins across several pathways. Experimental conditions were as outlined in Figure 2. (**A**) Volcano plot comparing phosphoprotein intensity between C5a-treated and control-treated cells, before phagocytosis. Proteins with adjusted *P* values less than 0.05 are shown in blue and proteins of interest (discussed in text) are labeled. *P* values were computed by limma-based linear models with Bonferroni’s correction for multiple testing. (**B**) Metascape (40) enrichment heatmap showing functional clusters of phosphoproteins dephosphorylated (DP) and phosphorylated (P) after C5a treatment.

Figure 3A shows a volcano plot comparing neutrophils treated with C5a versus vehicle control. 119 proteins were significantly differentially phosphorylated at 1 hour, indicating persistent signaling, consistent with the prolonged inhibition of phagocytosis seen in Figure 1. Notably, C5aR1 remained highly phosphorylated (a modification key to its internalization) (36), and this change has been used to identify C5a-exposed, dysfunctional neutrophils (2, 12, 13, 37–39). Pathway enrichment using Metascape (40) indicated involvement of pathways including membrane trafficking, regulated exocytosis (degranulation), and phosphatidylinositol-3,4,5-trisphosphate (PIP3) signaling, which persist 1 hour after stimulation with C5a (Figure 3B). Given the finding of a temporal dependency of C5a exposure on phagocytosis (Figure 1F), we examined how the phosphoproteomic alterations after C5a compared with those after *S*. *aureus*. The pathways showing prolonged activation after C5a exposure overlap with those induced by *S*. *aureus* (Supplemental Figure 4A), and many of the proteins are common to both conditions (Supplemental Figure 4B).

### S. aureus induces a marked alteration in the phosphoproteome, which is significantly affected by C5a exposure.

**Figure 4:**
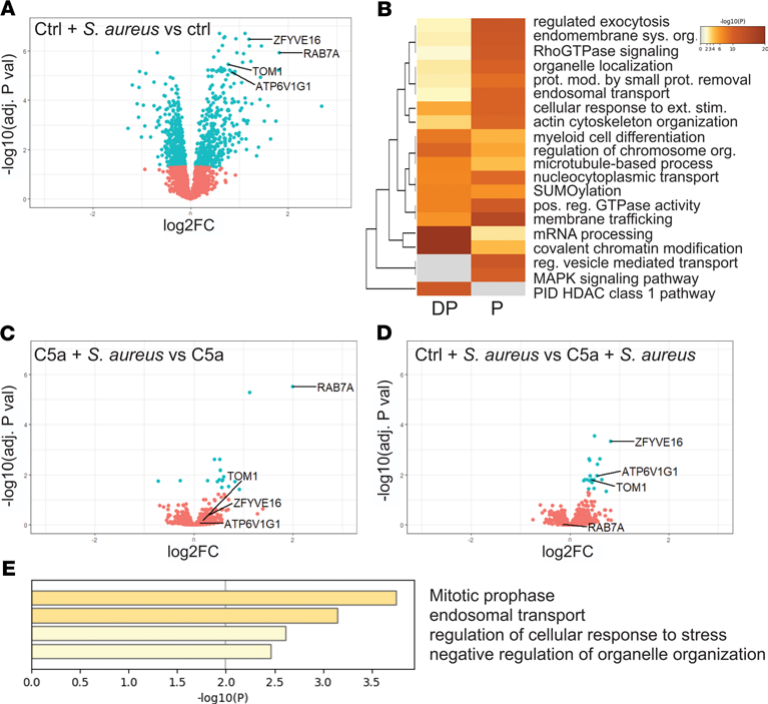
*S*. *aureus* induces a marked alteration in the phosphoproteome that is significantly affected by C5a exposure. Experimental conditions were as outlined in Figure 2. (**A**, **B**, and **D**) Proteins with adjusted *P* values less than 0.05 are shown in blue and proteins of interest (discussed in text) are labeled. Volcano plots compare phosphoprotein intensity between the conditions specified in the figure. *P* values were computed by limma-based linear models with Bonferroni’s correction for multiple testing. (**C**) Metascape (40) enrichment heatmap showing functional clusters of phosphoproteins dephosphorylated (DP) or phosphorylated (P) after *S*. *aureus* exposure. (**E**) Metascape (40) bar graph showing top nonredundant functional clusters of phosphoproteins enriched in the vehicle control/*S*. *aureus* condition versus C5a/*S*. *aureus* condition.

Exposure of neutrophils to *S*. *aureus* induced a marked alteration in the phosphoproteome (Figure 4A); 863 proteins (31% of the phosphoproteome) significantly alter their phosphorylation status. Pathway enrichment indicated the involvement of multiple pathways, notably Rho-GTPase signaling, endosomal transport, degranulation, and actin cytoskeleton organization (Figure 4B, with extended heatmap showing top 100 pathways shown in Supplemental Figure 5).

C5a exposure before *S*. *aureus* reduced the phosphoprotein response to the bacterium considerably (Figure 4C). However, comparing C5a and control treated cells exposed to *S*. *aureus*, 19 proteins were identified, suggesting selective pathway modulation (Figure 4D). When mapped to known pathways using Metascape (40) and manually annotated from the Uniprot database (41), a pattern of reduced phosphorylation of phagosomal maturation proteins (Table 1) and pathways (Figure 4E) emerged. Notably, early endosomal marker ZFYVE16 and its interactor TOM1 had impaired phosphorylation after C5a exposure, as did V-type ATPase subunit G1 (ATP6V1G1, which is critical for phagosomal acidification). ZFYVE16 requires phosphatidylinositol-3-phosphate (PI3P) for recruitment to the phagosome (42). Another prominent PI3P-responsive protein noted was Ras-related protein 7a (RAB7A), although this protein was not differentially phosphorylated between the C5a/*S*. *aureus* and vehicle control/*S*. *aureus* conditions. Supplemental Figure 6 shows individual donor data for these key proteins.

Our data set suggests that C5a exposure that precedes pathogen encounter prevents effective signaling through the phagosomal maturation pathways and links intracellular signaling to the prolonged functional impairment noted in this context. The other major cluster of differentially phosphorylated proteins comprised nuclear and nuclear membrane proteins, many of which are involved in mitosis and nuclear envelope integrity.

### C5a induces an impairment in phagosomal acidification, distinct from the impairment in ingestion.

**Figure 5:**
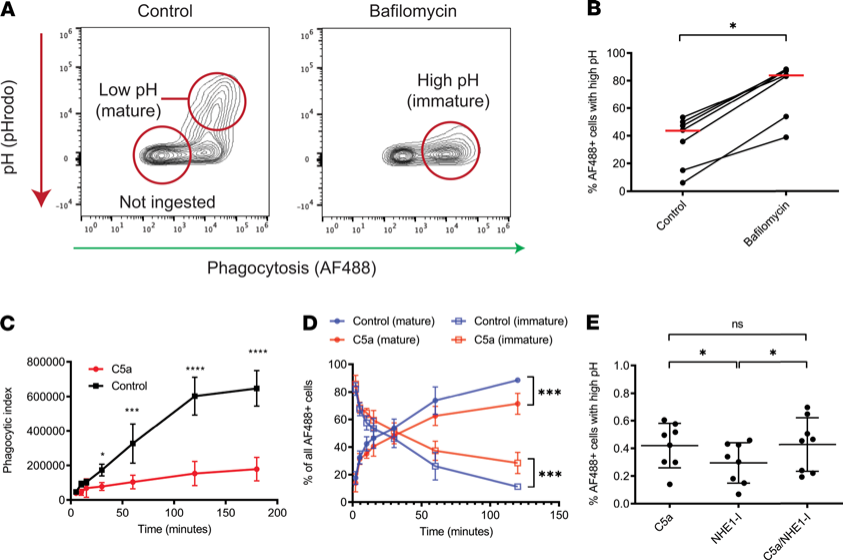
C5a induces an impairment in phagosomal acidification, distinct from the impairment in ingestion. (**A**) Exemplar flow cytometry plots of whole blood pretreated with vehicle control or bafilomycin A (100 nM, 60 minutes) before exposure to colabeled AF488/pHrodo red *S*. *aureus* (5 μg/mL) for 120 minutes. Both phagocytosis (x axis) and phagosomal pH (y axis) can be measured simultaneously in the same population of cells. pHrodo fluorescence increases with decreasing pH, indicating phagosomal maturity as shown. (**B**) Conditions as in A. Data are shown as individual data points with mean for *n* = 7 individual donors. *P* = 0.016 by Wilcoxon’s test. (**C**) Whole blood was pretreated with vehicle control or C5a (300 nM, 60 minutes) before exposure to phagocytosis probe (5 μg/mL) for 180 minutes. Phagocytosis without maturation (i.e., AF488 signal) is shown. Data are shown as mean plus or minus SD of *n* = 5 individual donors. ****P < 0.0001 by 2-way repeated-measures ANOVA with Bonferroni’s multiple comparisons test. (**D**) Conditions as in C. The percentage of *S*. *aureus* particle positive (AF488+) cells with low pH (mature) and high pH (immature) phagosomes is shown for control and C5a-treated conditions. Data are shown as mean plus or minus SD of *n* = 5 individual donors. ***P < 0.001 by repeated-measures 2-way ANOVA with Bonferroni’s multiple comparisons test. (**E**) Whole blood was pretreated with C5a (300 nM, 60 minutes), NHE-1 inhibitor (5 μM), or both, then exposed to maturation probe for 60 minutes. The percentage of AF488+ cells with high pH (immature) phagolysosomes is shown. Data are shown as mean plus or minus SD from *n* = 7 individual donors. *P* = 0.0080 by Friedman’s test, *P < 0.05 for Dunn’s test of multiple comparisons, ns = nonsignificant.

The phosphoproteomic signature of altered phagosomal maturation afterC5a exposure and the involvement of V-ATPase suggested that C5a had effects beyond impaired ingestion of bacteria. To disentangle the effects of phagocytic ingestion and phagolysosomal acidification, *S*. *aureus* bioparticles colabeled with the pH-insensitive dye AF488 and pHrodo red were used. Neutrophils ingested particles and then subsequently acidified the phagosome, a process which could be ablated by the addition of the V-ATPase inhibitor bafilomycin (43) (Figure 5, A and B). The use of this dual-labeled probe in time series experiments demonstrated that the processes of ingestion and phagosomal acidification are distinct (Supplemental Figure 7A). Addition of the weak base ammonium chloride at the end of the assay reduced the pHrodo signal, confirming the indicator measured changes in phagosomal pH independently of particle internalization (Supplemental Figure 7B), whereas live cell imaging demonstrated a progressive increase in pHrodo signal with time as phagosomes were acidified without further particle ingestion (Supplemental Figure 7, C–E, and Supplemental Video 1). C5a pretreatment increased the proportion of neutrophils that failed to ingest particles (Figure 5C) and increased the population that ingested particles but failed to acidify the phagosome (Figure 5D). Recent reports suggest that C5a induces Na^+^/H^+^ exchanger-1–mediated (NHE-1-mediated) cytoplasmic alkalinization (21). An NHE-1 inhibitor did not alter the C5a-mediated effect on phagosomal acidification (Figure 5E), suggesting that the pathways mediating these 2 effects of C5a on neutrophils are distinct. Furthermore, we confirmed previous work (14) showing C5a impaired ROS production (Supplemental Figure 8), which in combination with the current findings, suggests C5a induces a generalized failure of phagosomal maturation in addition to its effect on phagocytic ingestion.

### VPS34 inhibition impairs phagosomal acidification.

**Figure 6:**
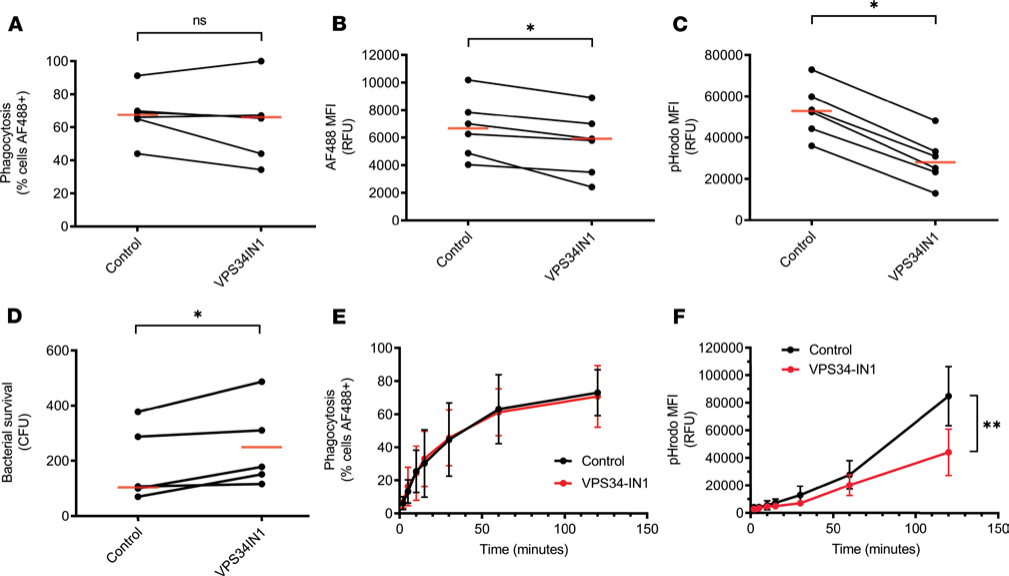
VPS34 inhibition impairs phagosomal acidification. Whole blood was pretreated with vehicle control or VPS34IN1 (1 μM, 60 minutes) before addition of maturation probe (5 μg/mL) (**A–D**), or live *S*. *aureus* (**E**), for 120 minutes before analysis. (**A**) Percentage of neutrophils that phagocytosed bioparticles. *P* = 0.31 by Wilcoxon’s test. *n* = 6 individual donors. (**B**) MFI of ingested particles, indicating relative quantity of phagocytosis. *P = 0.03. by Wilcoxon’s test. *n* = 6 individual donors. (**C**) pHrodo MFI, indicating phagosomal acidification. *P = 0.03. by Wilcoxon’s test. *n* = 6 individual donors. (**D**) After phagocytosis of live bacteria, human cells were lysed in alkaline dH_2_O and surviving bacteria were incubated overnight on blood agar. Bacterial survival was quantified by counting colonies. *P = 0.03 by paired *t* test, *n* = 5 individual donors. (**E** and **F**) Whole blood was processed as above with quantification of phagocytosis (**E**) and acidification (**F**) at the indicated time points. There was a reduction in phagosomal acidification as shown but no change in percentage of cells that underwent phagocytosis. Data are shown as mean plus or minus SD of *n* = 5 individual donors. **P = 0.0058 for drug treatment by 2-way repeated-measures ANOVA with Bonferroni’s multiple comparisons test.

The differential phosphoprotein analysis (Table 1) and phagosomal acidification assays (Figure 5) demonstrated impaired phagosomal maturation after exposure to C5a. As noted, several of the phosphoproteins that were differentially phosphorylated are known interactors with PI3P. The PI3K VPS34 is the dominant source of PI3P in mammalian cells (44). Although VPS34 itself was detected, its phosphorylation status was not significantly altered. However, the finding that C5a altered the phosphorylation status of PI3P-responsive proteins led us to explore the role of VPS34 in phagosomal acidification. We used the selective inhibitor, VPS34IN1 (45), to examine the role of this enzyme in phagosomal acidification, and how this related to the defect induced by C5a. VPS34IN1 did not alter the percentage of neutrophils that underwent phagocytosis (Figure 6A and time course in Figure 6E), but it did lead to a reduction in the overall number of particles ingested (Figure 6B) and a more marked reduction in pHrodo signal (Figure 6C and time course in Figure 6F), indicating VPS34IN1 impairs phagosomal acidification. VPS34 inhibition also led to an impairment in the killing of *S*. *aureus* (Figure 6D), similar to that observed with C5a (Supplemental Figure 1D) but without a significant reduction in phagosomal ROS production (Supplemental Figure 9).

### Neutrophils from critically ill patients exhibit defective phagosomal acidification.

**Figure 7:**
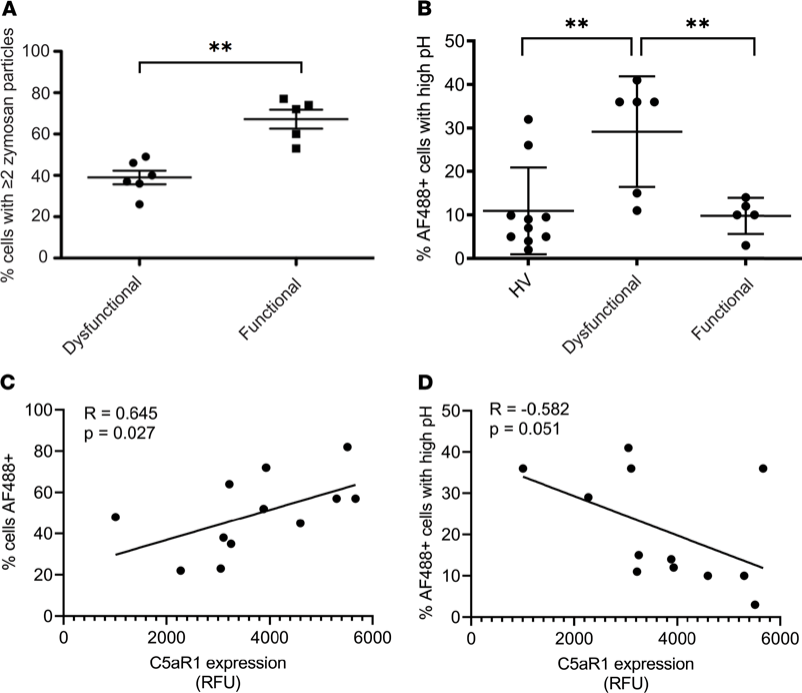
Neutrophils from critically ill patients exhibit defective phagosomal acidification. (**A**) Neutrophils purified from critically ill patients were exposed to zymosan (5 μg/mL) and allowed to phagocytose for 30 minutes as described in Methods and classified as functional or dysfunctional based on a cutoff of 50% of cells phagocytosing greater than or equal to 2 zymosan particles as previously described (12, 13). Data are shown as mean plus or minus SD for *n* = 6 patients with dysfunctional neutrophils and 5 patients with functional neutrophils respectively. **P = 0.004 by Mann-Whitney *U* test. (**B**) Neutrophil phagosomal acidification was assessed in whole blood from critically ill patients using the maturation probe as previously described. Patients were classed as dysfunctional using the assay from **A**. Data are shown as mean plus or minus SD *n* = 6 patients with dysfunctional neutrophils, 5 patients with functional neutrophils, and 10 healthy controls, respectively. *P* = 0.04 by 1-way ANOVA. **P < 0.01 by Holm-Šidák’s test of multiple comparisons. (**C** and **D**) C5aR1 expression was assessed by flow cytometry and correlated (Spearman) with phagocytosis (**C**) and phagosomal acidification (**D**) for *n* = 12 patients. NB: One patient’s cells did not adhere to tissue culture plastic for the zymosan assay, and thus they could not be assigned to dysfunctional or nondysfunctional groups shown in **A** and **B**. C5aR1 expression and maturation probe data was available to allow inclusion in correlation analyses in **C** and **D** and hence the difference in numbers between these figures.

To establish the relevance of our findings to the clinical setting, we used our assay of phagosomal acidification to interrogate neutrophils obtained from critically ill patients and healthy volunteers. We assessed neutrophil function in critically ill patients, defining neutrophil dysfunction as phagocytosis of less than 50% in our previously established zymosan assay (Figure 7A), a threshold associated with a markedly increased risk of nosocomial infection (12, 13, 46). Reduced internalization of *S*. *aureus* particles was also evident in patients with dysfunctional neutrophils identified in this manner (Supplemental Figure 10). Using our phagosomal acidification assay, we compared patients with dysfunctional neutrophils with critically ill patients with functional neutrophils and healthy controls. Dysfunctional neutrophils exhibited a failure of phagosomal acidification (Figure 7B) that was not seen in patients with functional neutrophils. Consistent with previous work (2, 12), we observed a correlation between C5aR1 expression (decreased after C5a exposure) and phagocytosis (Figure 7C) and an inverse correlation between C5aR1 expression and phagosomal acidification (Figure 7D), though the latter correlation did not reach statistical significance. The patients with dysfunctional and functional neutrophils could not be readily identified by clinical factors such as severity of illness or precipitating insult (Supplemental Table 2). These data provide evidence of dysfunctional phagosomal acidification in critically ill patients and imply a role for C5a in driving this dysfunction.

## Discussion

Our data demonstrate that C5a induces both a prolonged defect in phagocytosis of relevant pathogens (*S*. *aureus* and *E*. *coli*) and a persistent signaling across multiple pathways for some hours after the well characterized initial signaling events such as ionized calcium flux (47) and PIP3 generation (48). This finding supports the proposal that persistent C5a-induced signaling may mediate the neutrophil dysfunction observed in critically ill patients (12, 13).

To our knowledge, the data presented here (Figure 3, Figure 4, and Figure 5) represent the deepest assessment of the human neutrophil proteome and phosphoproteome (49–51). These data provide a phosphoproteomic assessment of the human neutrophil response to *S*. *aureus* and C5a. Unlike transcriptomic data (52–54), phosphoproteomics provides a direct assessment of mediators that are likely to have functional implications, especially in short-lived cells such as neutrophils (33, 55) and early pathogen exposure time points, as examined in this study.

The marked changes observed in phosphoproteins in response to *S*. *aureus* are perhaps unsurprising, as the response to and clearance of bacteria are primary functions of neutrophils. Many of the pathways identified (Figure 4 and Supplemental Figure 4) are consistent with established literature on neutrophil responses to *S*. *aureus*, and indeed other bacteria, including activation of PI3K (56), toll-like receptor signaling (57), and neutrophil degranulation (58).

The enrichment of PI3K and Rho GTPase signaling on C5a stimulation are in keeping with our previous identification of key roles for these molecules in C5a-mediated functional deficits in neutrophils (12, 13, 59). The marked suppression of the phosphorylation response to *S*. *aureus* induced by C5a pretreatment is not simply a response to reduced particle ingestion. Fifteen minutes after pathogen contact, there were limited differences in the ingestion rates between C5a and control treatments, and these became more marked over time (Figure 1). Furthermore, the differential analysis of C5a/*S*. *aureus* versus vehicle control/*S*. *aureus* conditions identified defects in specific signaling pathways, most notably those involving endosomal trafficking. This led us to examine the process of phagosomal maturation and to identification of a C5a-induced failure of phagosomal acidification (Figure 5) with similar findings in critically ill patients (Figure 7). Neutrophil phagolysosomes have been demonstrated to undergo a biphasic change in pH with early alkalinization at 3–5 minutes providing optimal conditions for protease activation (60–62), followed by progressive acidification. Our data are consistent with the later acidification demonstrated by these studies, though we did not specifically assess early time points with probes calibrated to measure exact pH. Failure of phagosomal maturation and intracellular killing has been described in primary immune deficiency (63), but it has not previously been described as part of the immunoparesis of critical illness. Impaired phosphorylation in pathways involving nuclear envelope breakdown and nuclear pore disassembly by C5a was unanticipated. The functional relevance of these changes remains unclear, though they may be early processes in the formation of nonlethal DNA-containing neutrophil extracellular traps (64).

Important signaling proteins involved in the process of phagosomal maturation (such as RAB7A, TOM1, and ZFYVE16) can be recruited to the phagosomal membrane by PI3P produced predominantly by VPS34 (42, 65, 66). Both ZFYVE16 and TOM1 phosphorylation were impaired by C5a exposure. We investigated the role of VPS34 as a mediator of neutrophil bactericidal function and found that selective VPS34 inhibition produced a similar impairment in phagosomal acidification to that observed with C5a (Figure 6). The finding that a similar defect could be induced by inhibiting VPS34, the dominant source of PI3P in neutrophils (44), adds further validation to the pathway signature identified in the phosphoproteomic profile.

Ellson and colleagues (67) demonstrated that PI3P plays an important role in targeting neutrophil oxidase components to phagosomal membranes, and its importance in phagosomal maturation has also been identified in *Dictyostelium discoideum* (68), murine macrophages, and macrophage-like cell lines (69). However, the role of VPS34 in human neutrophils has previously been inferred indirectly (70), owing to prior lack of selective inhibitors and the difficulties of genetically manipulating human neutrophils. Anderson and colleagues (70) demonstrated a role for VPS34 in NADPH oxidase–mediated ROS generation in neutrophils. We found a nonsignificant reduction in ROS production (Supplemental Figure 9) that was much less marked than the effect on phagosomal acidification. The reasons for these divergent findings are uncertain, though they may include differences in ROS measurement assays, our use of a selective VPS34 inhibitor, and differences between primary human neutrophils and cell lines. The mechanism by which VPS34 inhibition impairs killing of *S*. *aureus* requires further investigation, as phagosomal acidification is not thought to be critical to this process (71) and it is likely that the enzyme inhibition leads to further defects in phagosomal maturation. This study is unable to identify whether C5a prevents phagosomal acidification by inhibiting the recruitment of ATP6V1G1 to the phagosome or by preventing its function. Our data demonstrated that phosphorylation of the S68 residue of the V-ATPase G subunit was impaired by C5a pretreatment, and we hypothesize this posttranslational modification is critical to its ability to effectively acidify the phagosome. Further work is ongoing to address these issues.

It is intriguing to note that although VPS34 inhibition does not reduce the percentage of cells that phagocytose bacteria (Figure 6A), consistent with previous work (70), it does reduce the number of particles ingested (Figure 6B). This is consistent with the finding that ingestion of an initial target facilitates ingestion of a subsequent target (Supplemental Figure 2), implying a capacitive stage of phagocytosis. That VPS34 inhibition impairs this capacitive phase suggests a hitherto undescribed relationship between phagosomal maturation and the capacity of cells to ingest particles.

Our data also demonstrate that the timing of C5a exposure (before, alongside, or after pathogen encounter) has an important effect on neutrophil function. Only preexposure to C5a impaired subsequent neutrophil phagocytosis (Figure 2). Reduced C5aR1 availability for ligation by C5a is unlikely to explain this observation, as C5aR1 downregulation is induced by multiple agents that do not have the same effect on phagocytosis (Supplemental Figure 1D). The pathways enriched under C5a conditions are also represented among those enriched after *S*. *aureus* exposure, with all but 8 of the C5a-induced, significantly altered phosphoproteins also being identified after *S*. *aureus* exposure. It is therefore plausible that signaling induced by *S*. *aureus* overwhelms C5a-induced phosphorylation events unless they were established before *S*. *aureus* exposure. Indeed, the signaling induced by initial ingestion may facilitate subsequent ingestion, as identified in the sequential exposure assay (Supplemental Figure 2), actively mitigating against C5a-induced suppression. We did not identify a dose at which C5a enhanced phagocytosis in this work (Supplemental Figure 1A), indeed, nor did we in our previous work (12, 13). Collectively, these findings imply that the divergent findings on the effect of C5a on phagocytosis when C5a is added exogenously, as in this report or generated by direct addition of bacteria to blood as reported by Mollnes and colleagues (19, 20), are due to the temporal relationship between exposures rather than a biphasic dose–response relationship.

This finding of distinct temporal responses to C5a suggests that where neutrophils encounter bacteria and C5a at the same time, such as at the site of infection, the phagocytic response is not impaired. When complement activation spills over systemically and C5a exposure precedes neutrophil–bacterial interactions — as occurs with systematic inflammation in critical illness and sepsis — dysfunction occurs, impairing the host’s ability to respond to invasive infections or subsequent bacterial insults. (2, 38)

This study was conducted entirely in primary human neutrophils, using C5a an established, clinically relevant modulator of neutrophil function that has been linked to a range of adverse outcomes in critically ill patients. The use of clinically relevant pathogens and the development of a whole-blood bacteremia model increase the relevance of our study to the in vivo situation. Impaired ingestion of zymosan by neutrophils in patient has been associated with adverse outcomes including development of subsequent nosocomial infection (13). The finding that patients with such impairment also manifest impaired phagosomal acidification that correlates with markers of C5a exposure (Figure 7) and suggests that the identified mechanisms may be clinically relevant.

Several potential limitations should be highlighted. The phosphoproteomic response to *S*. *aureus* was evoked with heat-killed bacterial particles, conjugated with fluorescent dyes, and these may not fully reflect the response to live bacteria, although they do allow parallel functional assessment and standardization of the stimulus between donors and across research sites. Although whole blood is more physiologically relevant than cell culture media, it remains an abstraction from the situation in vivo, as it must be anticoagulated and does not involve normal flow or interaction with a vascular endothelium. Furthermore, the model may not reflect the function of neutrophils that have migrated into tissues, where most bacterial infections occur. Technical limitations currently prevent efficient phosphoproteomic assessment of cells from whole blood, and therefore isolated cells with the inherent in vitro artefacts must be used.

In conclusion, we have demonstrated the role of C5a in mediating neutrophil dysfunction in the clinically relevant setting of *S*. *aureus* and *E*. *coli* bacteremia, and demonstrated that the effects of C5a can persist for many hours and which is dependent on the temporal sequence of C5a and bacterial exposure. We also describe the neutrophil phosphoproteomic response to *S*. *aureus*, and to prolonged exposure to C5a. This approach identified a defective phagosomal maturation signature induced by C5a, likely involving modulation of Class III PI3K-dependent pathways. Further, we have shown the functional manifestation of this phosphorylation signature in a model of bacteremia. Finally, the clinical relevance of this failure of phagosomal acidification was observed in critically ill patients. A deeper understanding of the biology of neutrophil dysfunction in critical illness is key to developing effective treatments for a phenomenon associated with multiple adverse clinical outcomes.

## Methods

Supplemental Methods are available online with this article.

### Patients and healthy donors.

Patients were included if they were receiving support of one or more organ systems in an intensive care unit and expected to require such support for a further 24 hours and not thought likely to die within the next 24 hours. Exclusions were pregnancy, HIV infection, hematological malignancy, or immunosuppressive medication.

### Neutrophil isolation.

Neutrophils were isolated from citrated peripheral venous blood by using a modification of the discontinuous plasma/Percoll density gradient centrifugation technique initially described by Böyum in 1968 (72). Further details of methods and reagents described below are available in the supplementary materials.

### Phagocytosis of pHrodo S. aureus and E. coli bioparticles by purified neutrophils.

Purified human neutrophils, suspended in Iscoves Modified Dulbecco’s Medium (IMDM; Life Technologies) with 1% autologous serum at a concentration of 5 x 10^6^/mL were incubated in microcentrifuge tubes (Eppendorf) with purified human C5a (Complement Technology) or vehicle control. pHrodo-conjugated *S*. *aureus* or *E*. *coli* bioparticles (Life Technologies) were opsonized in 50% autologous serum for 30 minutes before be being added to the suspended cells. Analysis was by flow cytometry (Attune NxT, Thermo Fisher Scientific)

### No-wash, no-lyse whole blood assay of neutrophil phagocytosis and ROS production.

Blood, collected into argatroban (150 μg/mL, R&D Systems), was treated with inhibitors or priming agents, as indicated in the respective figure legends (Figures 1–3 and 5 and Supplemental Figures 1, 3, and 8), before being exposed to *S*. *aureus* pHrodo/dihydrorhodamine (CalBiochem) or *E*. *coli* pHrodo bioparticles. Aliquots were stained on ice with anti-CD16 antibody (clone 3G8, BioLegend) diluted and analyzed by flow cytometry (Attune NxT). Where reported, the phagocytic index is the median fluorescence intensity multiplied by the percentage of phagocytosing cells. The concentration of bioparticles used in each experiment is specified in Figures 1, 2, and 5–7 and Supplemental Figures 1, 3, and 7.

In variations on this assay, *S*. *aureus* particles labeled with the pH-insensitive dye AlexaFluor AF488 (Life Technologies) or dual labeled with AF488 and pHrodo red were used. pHrodo red conjugation of AF488 *S*. *aureus* was performed in-house using the pHrodo Particle Labeling Kit (Thermo Fisher Scientific). Fluorescence of extracellular particles was quenched with trypan blue (0.1mg/mL, Life Technologies). For experiments with inhibitors including C5a, VPS34IN1, and bafilomycin, the inhibitors were added at the concentrations noted in the figure legends for Figures 1–3, 5, and 6 and Supplemental Figures 1, 3, 8, and 9 for the duration indicated, before exposure to *S*. *aureus*. For ammonium chloride phagosomal neutralization, cells were exposed to pHrodo red-labeled particles for the duration indicated in Supplemental Figure 7, then exposed to 15 mM ammonium chloride for 5 minutes before analysis by flow cytometry.

Patient samples, and a comparator group of healthy donors, were analyzed in a different laboratory that did not have access to an Attune Nxt flow cytometer; to fit with established workflows in this laboratory, red cells were lysed using Pharmlyse (BD Biosciences) followed by washing twice using a Facswash Assistant (BD Biosciences) before undertaking flow cytometry (Fortessa, BD Biosciences).

### Bacterial killing assay — whole blood.

Methicillin-sensitive *S*. *aureus* bacteria (strain ASASM6) were grown to early log phase. Blood was collected into argatroban and incubated with bacteria for 1 hour. Human cells were lysed by addition of pH 11 distilled water for 3 minutes before plating of serial dilutions on Colombia blood agar.

### Neutrophil phagocytosis of zymosan.

Purified human neutrophils (suspended in phenol red-free IMDM with 1% autologous serum at a concentration of 10^6^/mL) were allowed to adhere to 24-well tissue culture plates (Sarstedt) for 20 minutes. Opsonized zymosan (5 μg/mL, MilliporeSigma) was then added for 30 minutes, before noninternalized zymosan particles were washed off with warm IMDM. Plates were incubated at all stages at 37°C in 5% CO_2_. Plates were allowed to air dry before being fixed with methanol and stained with QuickDIFF staining kit (Reagena). Phagocytosis was measured by manual counting of cells under light microscopy. Neutrophils with greater than or equal to 2 internalized zymosan particles were counted as a proportion of total neutrophils in 2 separate fields per well as previously described (12).

### Preparation of whole human neutrophil lysates for phosphoproteomics.

Neutrophils were isolated from whole blood as detailed above and resuspended in RPMI 1640 media (Thermo Fisher Scientific) containing 10 Mm HEPES (Thermo Fisher Scientific) with 1% autologous serum at a concentration of 1 × 10^7^ cells/mL.

### Proteomic and phosphoproteomic studies.

Triplicates of 1 × 10^7^ neutrophils were treated with vehicle control or C5a (100 nM, 60 minutes) at 37°C before addition of pHrodo *S*. *aureus* (15 μg/mL). Phagocytosis was allowed to occur for 15 minutes. Aliquots were withdrawn from each triplicate and pooled at the indicated time points. Cells were centrifuged at 400 *g* for 5 minutes at 4°C, supernatants aspirated, and cell pellets were snap frozen in liquid nitrogen. Cells were lysed by the addition of sodium dodecyl sulphate (0.5%; MilliporeSigma) and triethylammonium bicarbonate buffer (0.1 M; MilliporeSigma) and sonicated, before undergoing centrifugation, trypsin digestion, tandem mass tag labeling, fractionation, phosphopeptide enrichment, and liquid chromatography and tandem mass spectrometry analysis. See Supplemental Figure 11 for details and experimental schematic.

### Statistical analysis of wet laboratory data.

Data are presented as individual data points with summary statistics (median ± IQR or mean ± SD) according to whether data are normally distributed. Parametric or nonparametric statistical tests were applied as appropriate after data were tested for normality using the Kolmogorov-Smirnov test. Tests used for comparisons are indicated in figure legends. Two-tailed *P* values were computed; a *P* value of less than 0.05 was considered statistically significant. Statistical analyses were undertaken using GraphPad Prism Software v8.0.

### Statistical analysis of phosphoproteomics data.

Spectral.raw files from data dependent acquisition were processed with the SequestHT search engine on Thermo Scientific Proteome Discoverer 2.1 software. Data were searched against both human and *S*. *aureus* UniProt reviewed databases at a 1% spectrum–level FDR criteria using Percolator (University of Washington, USA). MS1 mass tolerance was constrained to 20 ppm, and the fragment ion mass tolerance was set to 0.5 Da. TMT tags on lysine residues and peptide N termini (+229.163 Da), and methylthio (+45.988 Da) of cysteine residues (+45.021 Da) were set as static modifications, whereas oxidation of methionine residues (+15.995 Da) and deamidation (+0.984 Da) of asparagine and glutamine residues were set as variable modifications. For TMT-based reporter ion quantitation, we extracted the signal-to-noise ratio for each TMT channel. Parsimony principle was applied for protein grouping.

Peptide and phosphopeptide intensities were normalized across conditions using median scaling and then summed to generate protein and phosphoprotein intensities. Proteins and phosphoproteins were independently identified and quantified in all samples from all 4 donors; species not meeting these criteria were excluded from subsequent analysis. Log2 fold change was calculated between conditions of interest, compared across *n* = 4 donors, and tested for statistical significance by limma-based linear models with Bonferroni’s correction for multiple testing. Hierarchical clustering using Euclidean distance was undertaken on the entire data set. Heatmaps and volcano plots were generated as shown in Results. Statistical analyses were performed in RStudio (73) using the qPLEXanalyzer (35) package, and plots were produced using the ggplot2 (74) package.

### Statistics.

Tests used were 2-tailed paired *t* test for comparison of 2 conditions in the same donor where data were normally distributed, or Wilcoxon matched pairs for nonnormally distributed data from the same donor. Where 2 or more conditions were compared over time in the same donors repeated measures 2-way ANOVA was used with Dunnet’s, Holm-Sidak’s, or Bonferroni’s post hoc tests. For 2 or more conditions at a single timepoint from the same donors Friedman’s was used with Dunn’s post hoc test. For comparisons between 2 groups of donors Mann-Whitney *U* test was used, and for 3 conditions at a single timepoint from different donors 1-way ANOVA used with Bonferroni’s post hoc test. Correlations were assessed by Spearman’s rho. For phosphoproteomic intensity changes *P* values were calculated by limma-based linear modelling with Bonferroni’s correction for multiple analyses. normality testing was conducted using the Kolmogorov-Smirnov test.

### Study approvals.

Ethical permission for obtaining peripheral venous blood from healthy volunteers was provided by the Cambridge Local Research Ethics Committee (REC reference: 06/Q0108/281) and all donors provided written, informed consent. Critically ill patient blood samples were obtained under an approval granted by the North East-Newcastle & North Tyneside 2 Research Ethics Committee (REC reference: 18/NE/0036). Healthy volunteer blood samples for comparison with the critically ill patient samples were obtained under the approval granted by the County Durham and Tees Valley Research Ethics Committee (REC reference: 12/NE/0121).

### Data sharing statement.

The mass spectrometry proteomics data have been deposited to the ProteomeXchange Consortium via the PRIDE (75) partner repository with the data set identifier PXD017092.

## Author contributions

AJTW contributed conceptualization, formal analysis, investigation, methodology, validation, visualization, and writing (original draft, review, and editing). AMV contributed investigation, methodology, and writing (review and editing). MHRS contributed investigation, methodology, and writing (review and editing). JS contributed investigation, methodology, and writing (review and editing). CZ contributed investigation and writing (review and editing). CGT contributed investigation, methodology, and writing (review and editing). KK contributed methodology, data curation, software, formal analysis, and writing (review and editing). CSD contributed project administration, resources, supervision, and writing (review and editing). AJS contributed project administration, resources, supervision, and writing (review and editing). DKM contributed project administration, resources, supervision, and writing (review and editing). CS contributed methodology, validation, project administration, resources, supervision, and writing (review and editing). ERC contributed conceptualization, formal analysis, funding acquisition, methodology, project administration, resources, supervision, and writing (review and editing). KO contribtued project administration, resources, supervision, and writing (review and editing). ACM contributed conceptualization, formal analysis, funding acquisition, investigation, methodology, project administration, resources, supervision, validation, and writing (original draft, review, and editing).

## Supplementary Material

Supplemental data

Supplemental Video 1

## Figures and Tables

**Table 1:**
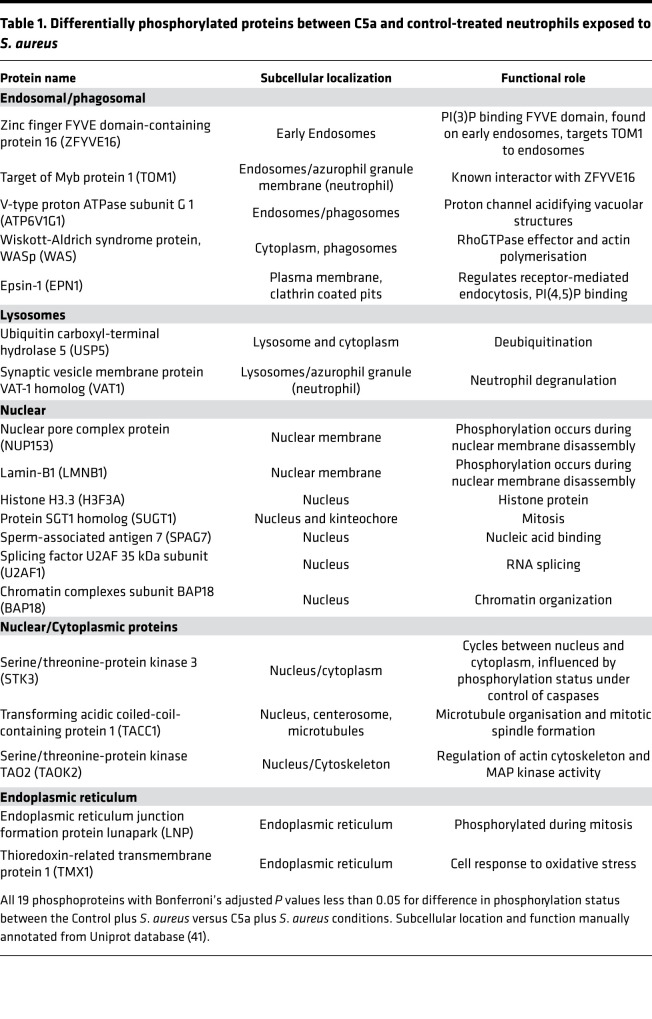
Differentially phosphorylated proteins between C5a and control-treated neutrophils exposed to *S. aureus*

## Version 1 07/07/2020

In-Press Preview

## Version 2 08/06/2020

Electronic publication
