# Supplementary material for: C5a impairs phagosomal maturation in the neutrophil through phosphoproteomic remodeling
Source: JCI Insight. 2020 Aug 6;5(15):e137029. doi: 10.1172/jci.insight.137029 (PMC7455072; doi:10.1172/jci.insight.137029)
Supplement: Supplemental data [file jciinsight-5-137029-s237.pdf]

# Supplemental Material

## Supplemental Table 1: Marked phosphoproteome changes with treatment

This table shows statistically significant numbers of protein/phosphoprotein alterations in the total proteome and total phosphoproteome between the conditions indicated. These alterations are shown as both the absolute number and as a percentage of the respective proteome/phosphoproteome.

| | Total proteome (4859 proteins quantified) | | Total phosphoproteome (2712 phosphoproteins quantified) | |
|---|---|---|---|---|
| Conditions compared | Number of proteins significantly altered | Percentage of total proteome significantly altered | Number of phosphoproteins significantly altered | Percentage of total phosphoproteome significantly altered |
| Ctrl-untreated | 0 | 0.0% | 16 | 0.6% |
| C5a-ctrl | 7 | 0.1% | 111 | 4.1% |
| *S. aureus*-ctrl | 95 | 2.0% | 858 | 31.6% |

**Supplemental Table 2: Clinical characteristics of critically ill patients who underwent neutrophil functional assessment**

| Parameter | Patients with Dysfunctional neutrophils (n=6) | Patients with non-dysfunctional neutrophils (n=5) |
|---|---|---|
| Median (range) age | 62 (44-77) | 65 (48-81) |
| % female (n) | 33% (2) | 20% (1) |
| Median (Interquartile range) admission APACHE II score | 19.5 (17.25-29) | 16.5 (12-21) |
| % with suspected sepsis on admission<br>(of which culture positive) | 66% (4)<br>100% (4) | 100% (5)<br>60% (3) |

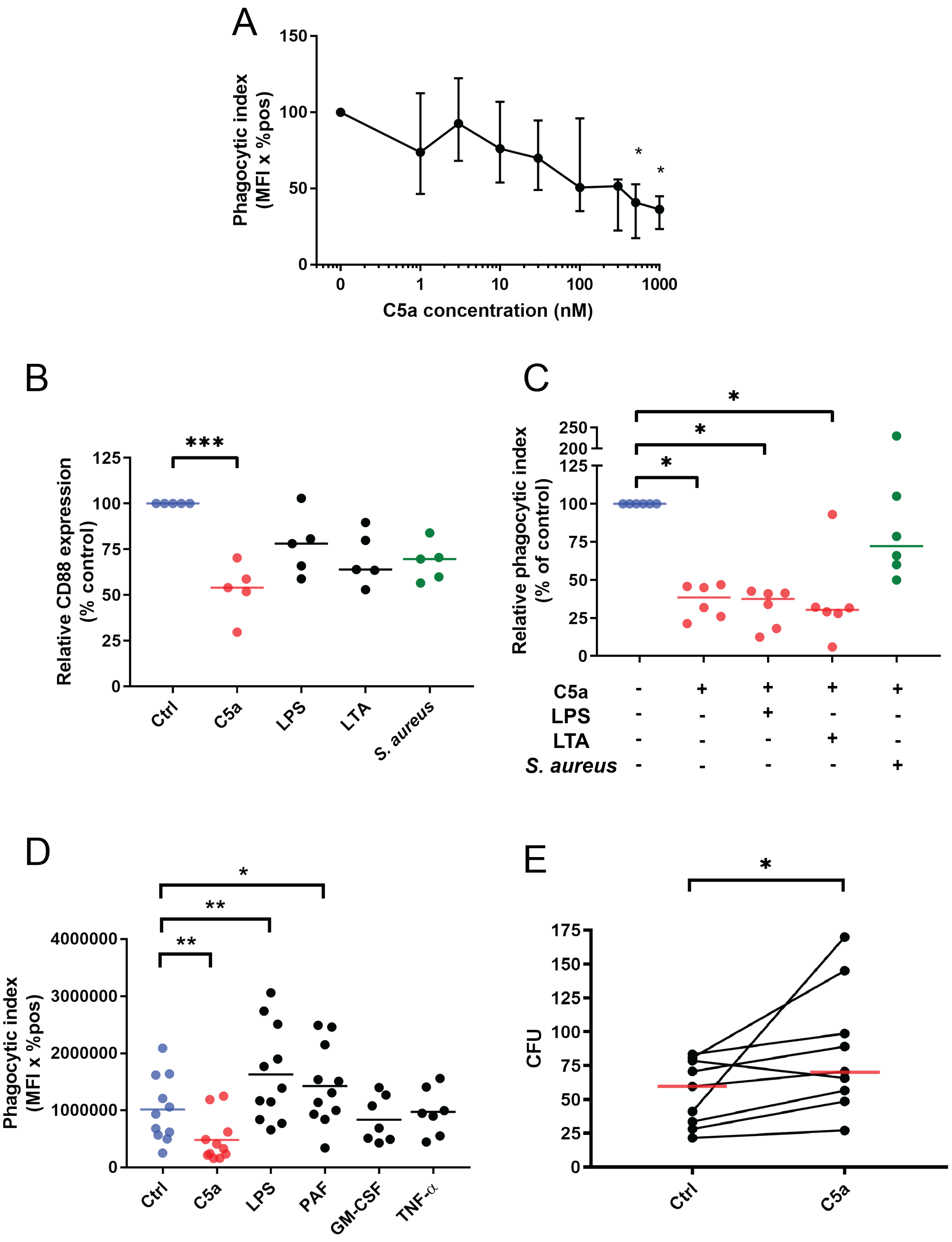

A
Phagocytic index (MFI x %pos)
C5a concentration (nM)
B
Relative CD88 expression (% control)
Ctrl
C5a
LPS
LTA
S. aureus
C
Relative phagocytic index (% of control)
C5a
LPS
LTA
S. aureus
D
Phagocytic index (MFI x %pos)
Ctrl
C5a
LPS
PAF
GM-CSF
TNF-α
E
CFU
Ctrl
C5a

## Supplemental figure 1: C5aR1 changes and phagocytosis with inflammatory stimuli, neutrophil killing of *S. aureus*

**A:** Whole blood was pre-treated with the indicated concentrations of C5a for 60 minutes prior to phagocytosis of pHrodo bioparticles. Data are shown as the median and IQR of 4 independent experiments relative to control-treated blood. Friedman p-value = 0.0024, $^*p < 0.05$ by Dunn's multiple comparisons.

**B**: Whole blood was incubated with the indicated inflammatory stimuli for 60 minutes prior to measurement of neutrophil C5aR1 expression by flow cytometry: 300 nM C5a; 100 ng/mL LPS; 10 µg/mL LTA; *S. aureus* bioparticles 10 µg/mL. Individual data points and their median values from n = 5 independent experiments are shown. Friedman's test with p-value = 0.0003, $^{***}p < 0.001$ by Dunn's multiple comparisons test.

**C**: Whole blood was pre-incubated with control or inflammatory stimuli as in (A) and then with 300 nM C5a for 60 minutes. Cells were then allowed to phagocytose 10 µg/mL *S. aureus* pHrodo™ red Bioparticles for 60 minutes before phagocytosis was assessed by flow cytometry as previously described. Individual data points and their median values from n = 6 independent experiments are shown. Friedman's test with p-value = 0.0038, $^*p < 0.05$ by Dunn's multiple comparisons test.

**D**: Whole blood neutrophil phagocytosis after pre-treatment with various agonists. Concentrations and pre-treatment durations were as follows: Ctrl (PBS) 60 minutes; C5a 300 nM, 60 minutes; LPS 100 ng/mL, 60 minutes; PAF 1 µM, 5 minutes; GM-CSF 10 ng/mL, 30 minutes; TNF-α 20 ng/mL, 30 minutes. ANOVA p-value < 0.0001 for both D and E, $^*p < 0.05$, $^{**}p < 0.01$ for Dunnett's multiple comparisons. Data are shown as individual data points with means for n = 11 (E) and n = 7 (F) individual donors.

**E**: Purified human neutrophils were pre-treated with 100 n C5a for 60 minutes prior to incubation with live *S. aureus* (strain ASASM6) for 60 minutes at an MOI of 10:1 bacteria per neutrophil. Neutrophils were lysed, bacteria plated and grown overnight and CFUs quantified

by visual inspection. Individual data points from n = 9 independent experiments are shown. C5a pre-treatment resulted in an increase in median CFU count of 30.8 % relative to control. $p = 0.02$ by Wilcoxon.

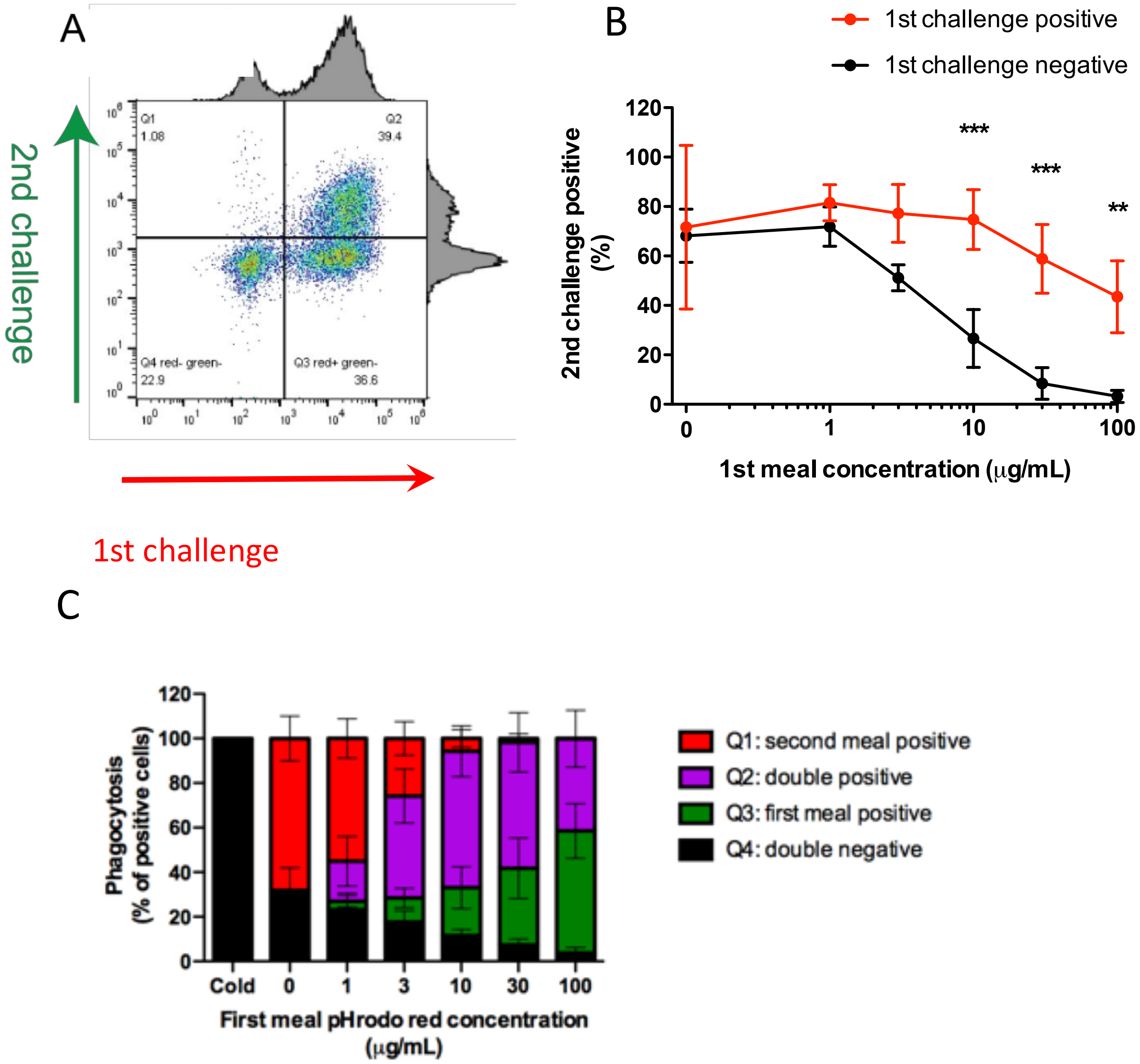
A
Q1
1.08
Q2
39.4
Q4 red- green-
22.9
Q3 red+ green-
36.6
2nd challenge
1st challenge
B
1st challenge positive
1st challenge negative
2nd challenge positive (%)
***
***
**
1st meal concentration (μg/mL)
C
Phagocytosis (% of positive cells)
Cold
0
1
3
10
30
100
First meal pHrodo red concentration (μg/mL)
Q1: second meal positive
Q2: double positive
Q3: first meal positive
Q4: double negative

**Supplemental figure 2: whole blood sequential 'feeding' assay indicates a facilitatory relationship between prior and subsequent ingestion of phagocytic targets.**

**A:** Example flow plot showing results from healthy donor blood exposed to 15µg/ml of pHrodo red *S. aureus* for 30 minutes followed 15µg/ml of pHrodo green *S. aureus* for 30 minutes. Cells divide into 3 populations, bottom left quadrant low/no phagocytosis, bottom right, single phagocytosis, top right double phagocytosis. Of note very few cells ingest the second meal having not ingested the first (top left quadrant).

**B:** Increasing the concentration of the first 'meal' reduces the proportion of cells eating a standard (15 µg/mL) sized second 'meal', indicating a fundamental limit on the capacity of neutrophils to ingest particles. Increasing the size of the first meal leads to progressive selection of a resolutely non-phagocytic population corresponding to Q1 shown in panel A. Data are shown as mean ± SD from n=5 individual donors, $P<0.0001$ by repeated measures two way ANOVA. *** $p<0.001$, **$P<0.01$ by Bonferroni's post-hoc test.

**C:** Proportions of cells in each quadrant with increasing size of first 'meal'. Data are shown as mean ± SD from n=5 individual donors

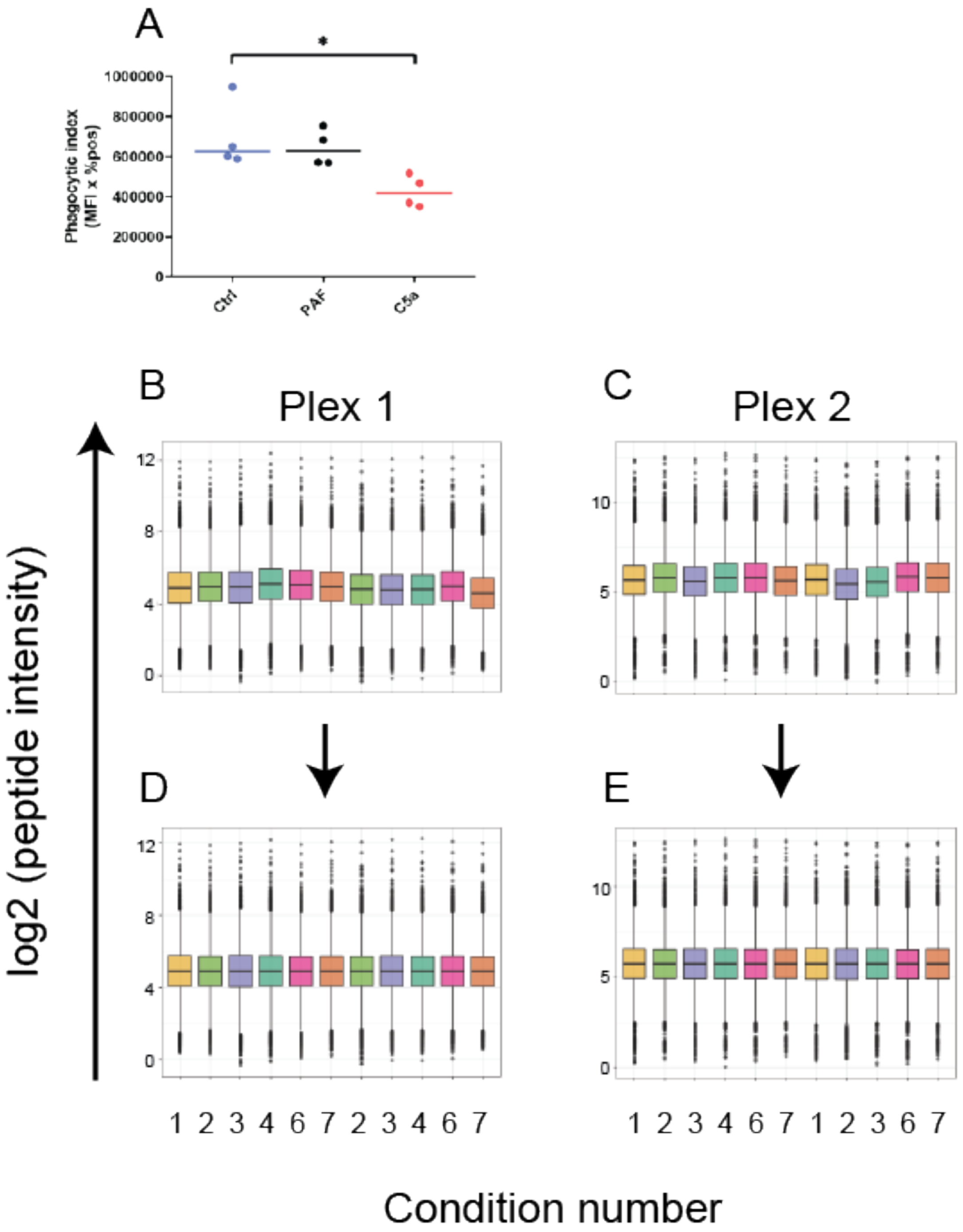

A
*
Phagocytic index (MFI x %pos)
1000000
800000
600000
400000
200000
0
Ctrl
PAF
C5a
B
Plex 1
C
Plex 2
D
E
log2 (peptide intensity)
12
8
4
0
10
5
0
1 2 3 4 6 7 2 3 4 6 7
1 2 3 4 6 7 1 2 3 6 7
Condition number

**Supplemental figure 3: technical validation of phosphoproteome assessment**

**A**: Neutrophils were pre-treated with 100 nM C5a for 60 minutes or 1 µM PAF for 5 minutes prior to phagocytosis of 15 µg/mL of *S. aureus* Bioparticles for 15 minutes. C5a pre-treatment led to a reduction in median phagocytosis of 33.2 % relative to control. Individual data points of each donor are shown with medians. Friedman p-value 0.042, $*p < 0.05$ by Dunn's multiple comparisons test.

**B-E**: Median, IQR and outliers of the measured peptide intensity for the 2712 quantified phosphoproteins are shown for each experimental condition on each plex. Intensities are shown before (A, B) and after (C, D) median scaling normalisation of data.

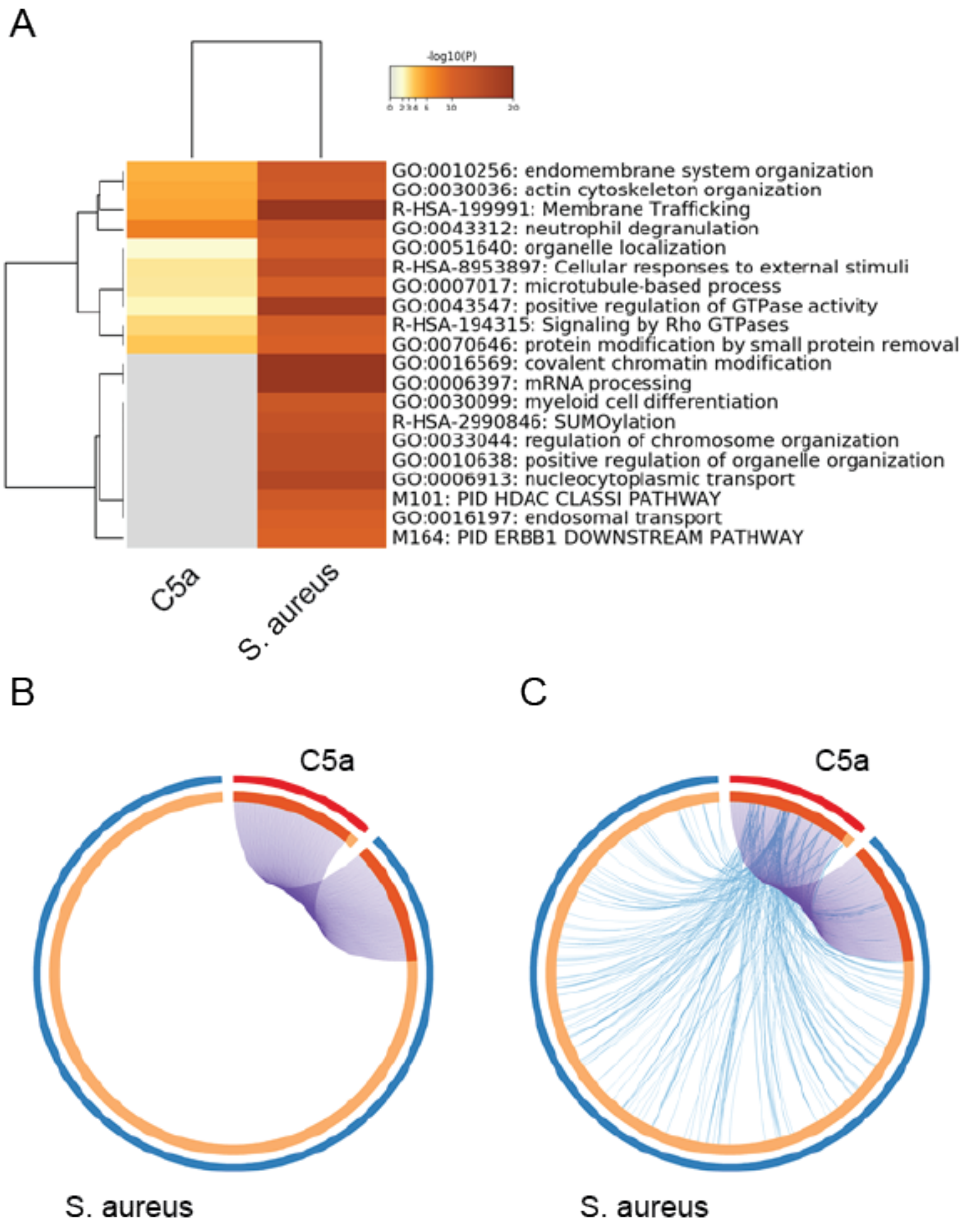
A
-log10(P)
GO:0010256: endomembrane system organization
GO:0030036: actin cytoskeleton organization
R-HSA-199991: Membrane Trafficking
GO:0043312: neutrophil degranulation
GO:0051640: organelle localization
R-HSA-8953897: Cellular responses to external stimuli
GO:0007017: microtubule-based process
GO:0043547: positive regulation of GTPase activity
R-HSA-194315: Signaling by Rho GTPases
GO:0070646: protein modification by small protein removal
GO:0016569: covalent chromatin modification
GO:0006397: mRNA processing
GO:0030099: myeloid cell differentiation
R-HSA-2990846: SUMOylation
GO:0033044: regulation of chromosome organization
GO:0010638: positive regulation of organelle organization
GO:0006913: nucleocytoplasmic transport
M101: PID HDAC CLASSI PATHWAY
GO:0016197: endosomal transport
M164: PID ERBB1 DOWNSTREAM PATHWAY
C5a
S. aureus
B
C5a
S. aureus
C
C5a
S. aureus

**Supplemental Figure 4: C5a induced changes in phosphoproteins which show overlap with changes induced by *Staph aureus.***

**A:** Pathway mapping by Metascape (40) demonstrates C5a-induced changes in phosphoproteins enrich pathways which are also enriched by phosphoprotein changes after *Staph. aureus* exposure.

**B, C**: Overlap diagram for individual phosphoproteins showing significant change under C5a and *S. aureus* conditions, where purple lines link proteins identified in both data sets (B) and including proteins linked at the shared term level where blue lines link those proteins which belong to the same enriched ontology term (C).

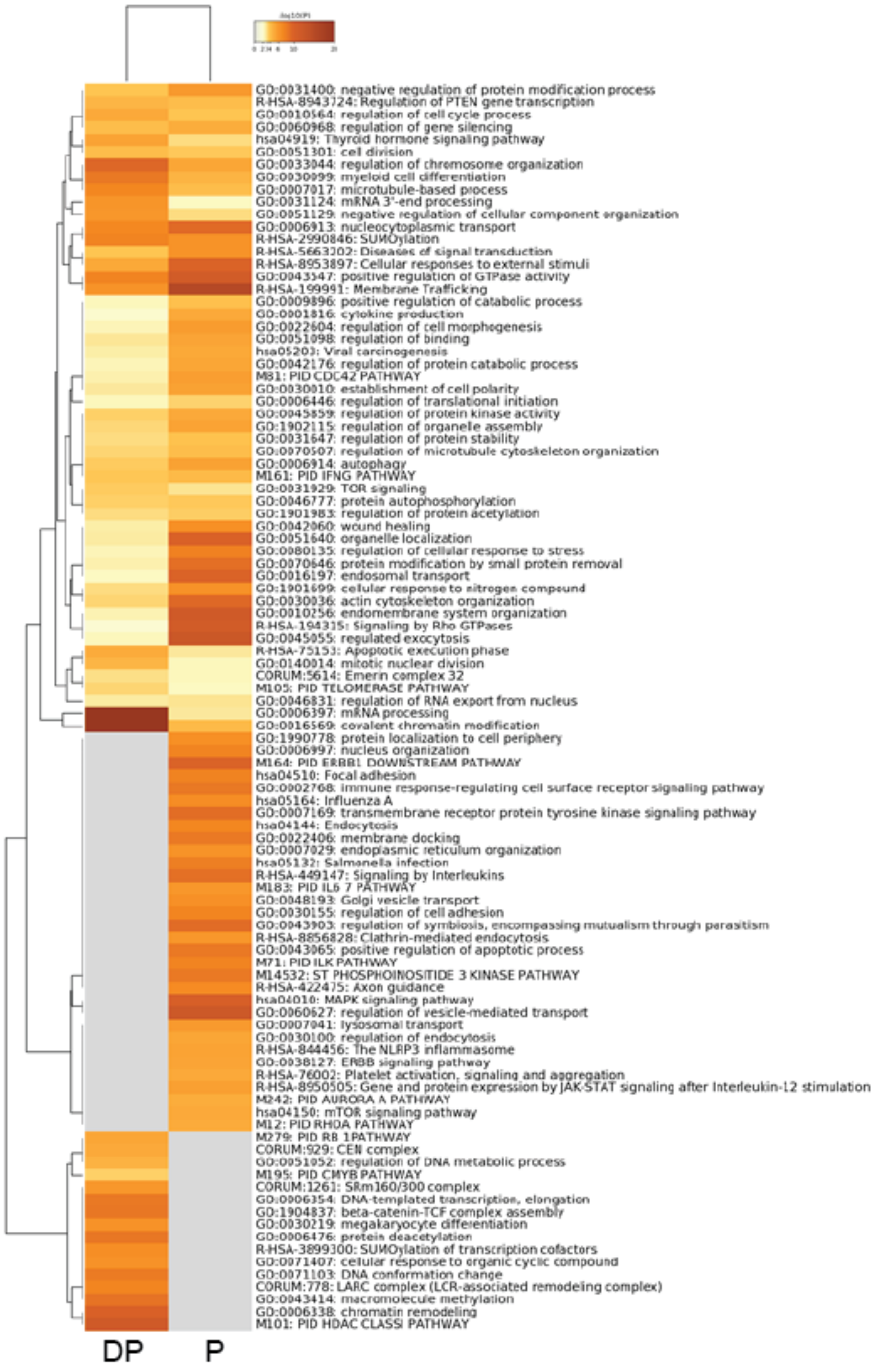
GO:0031400: negative regulation of protein modification process
R-HSA-8943724: Regulation of PTEN gene transcription
GO:0010564: regulation of cell cycle process
GO:0060968: regulation of gene silencing
hsa04919: Thyroid hormone signaling pathway
GO:0051301: cell division
GO:0033044: regulation of chromosome organization
GO:0030099: myeloid cell differentiation
GO:0007017: microtubule-based process
GO:0031124: mRNA 3'-end processing
GO:0051129: negative regulation of cellular component organization
GO:0006913: nucleocytoplasmic transport
R-HSA-2990846: SUMOylation
R-HSA-5663202: Diseases of signal transduction
R-HSA-8953897: Cellular responses to external stimuli
GO:0043547: positive regulation of GTPase activity
R-HSA-199991: Membrane Trafficking
GO:0009896: positive regulation of catabolic process
GO:0001816: cytokine production
GO:0022604: regulation of cell morphogenesis
GO:0051098: regulation of binding
hsa05203: Viral carcinogenesis
GO:0042176: regulation of protein catabolic process
M81: PID CDC42 PATHWAY
GO:0030010: establishment of cell polarity
GO:0006446: regulation of translational initiation
GO:0045859: regulation of protein kinase activity
GO:1902115: regulation of organelle assembly
GO:0031647: regulation of protein stability
GO:0070507: regulation of microtubule cytoskeleton organization
GO:0006914: autophagy
M161: PID IFNG PATHWAY
GO:0031929: TOR signaling
GO:0046777: protein autophosphorylation
GO:1901983: regulation of protein acetylation
GO:0042060: wound healing
GO:0051640: organelle localization
GO:0080135: regulation of cellular response to stress
GO:0070646: protein modification by small protein removal
GO:0016197: endosomal transport
GO:1901699: cellular response to nitrogen compound
GO:0030036: actin cytoskeleton organization
GO:0010256: endomembrane system organization
R-HSA-194315: Signaling by Rho GTPases
GO:0045055: regulated exocytosis
R-HSA-75153: Apoptotic execution phase
GO:0140014: mitotic nuclear division
CORUM:5614: Emerin complex 32
M105: PID TELOMERASE PATHWAY
GO:0046831: regulation of RNA export from nucleus
GO:0006397: mRNA processing
GO:0016569: covalent chromatin modification
GO:1990778: protein localization to cell periphery
GO:0006997: nucleus organization
M164: PID ERBB1 DOWNSTREAM PATHWAY
hsa04510: Focal adhesion
GO:0002768: immune response-regulating cell surface receptor signaling pathway
hsa05164: Influenza A
GO:0007169: transmembrane receptor protein tyrosine kinase signaling pathway
hsa04144: Endocytosis
GO:0022406: membrane docking
GO:0007029: endoplasmic reticulum organization
hsa05132: Salmonella infection
R-HSA-449147: Signaling by Interleukins
M183: PID IL6 7 PATHWAY
GO:0048193: Golgi vesicle transport
GO:0030155: regulation of cell adhesion
GO:0043903: regulation of symbiosis, encompassing mutualism through parasitism
R-HSA-8856828: Clathrin-mediated endocytosis
GO:0043065: positive regulation of apoptotic process
M71: PID ILK PATHWAY
M14532: ST PHOSPHOINOSITIDE 3 KINASE PATHWAY
R-HSA-422475: Axon guidance
hsa04010: MAPK signaling pathway
GO:0060627: regulation of vesicle-mediated transport
GO:0007041: lysosomal transport
GO:0030100: regulation of endocytosis
R-HSA-844456: The NLRP3 inflammasome
GO:0038127: ERBB signaling pathway
R-HSA-76002: Platelet activation, signaling and aggregation
R-HSA-8950505: Gene and protein expression by JAK-STAT signaling after Interleukin-12 stimulation
M242: PID AURORA A PATHWAY
hsa04150: mTOR signaling pathway
M12: PID RHOA PATHWAY
M279: PID RB 1PATHWAY
CORUM:929: CEN complex
GO:0051052: regulation of DNA metabolic process
M195: PID CMYB PATHWAY
CORUM:1261: SRm160/300 complex
GO:0006354: DNA-templated transcription, elongation
GO:1904837: beta-catenin-TCF complex assembly
GO:0030219: megakaryocyte differentiation
GO:0006476: protein deacetylation
R-HSA-3899300: SUMOylation of transcription cofactors
GO:0071407: cellular response to organic cyclic compound
GO:0071103: DNA conformation change
CORUM:778: LARC complex (LCR-associated remodeling complex)
GO:0043414: macromolecule methylation
GO:0006338: chromatin remodeling
M101: PID HDAC CLASSI PATHWAY
DP
P

**Supplemental Figure 5: Signalling pathway enriched by *S. aureus* exposure.**

Metascape (40) enrichment heatmap showing functional clusters of phosphoproteins dephosphorylated (DP) and phosphorylated (P) after *S. aureus* exposure.

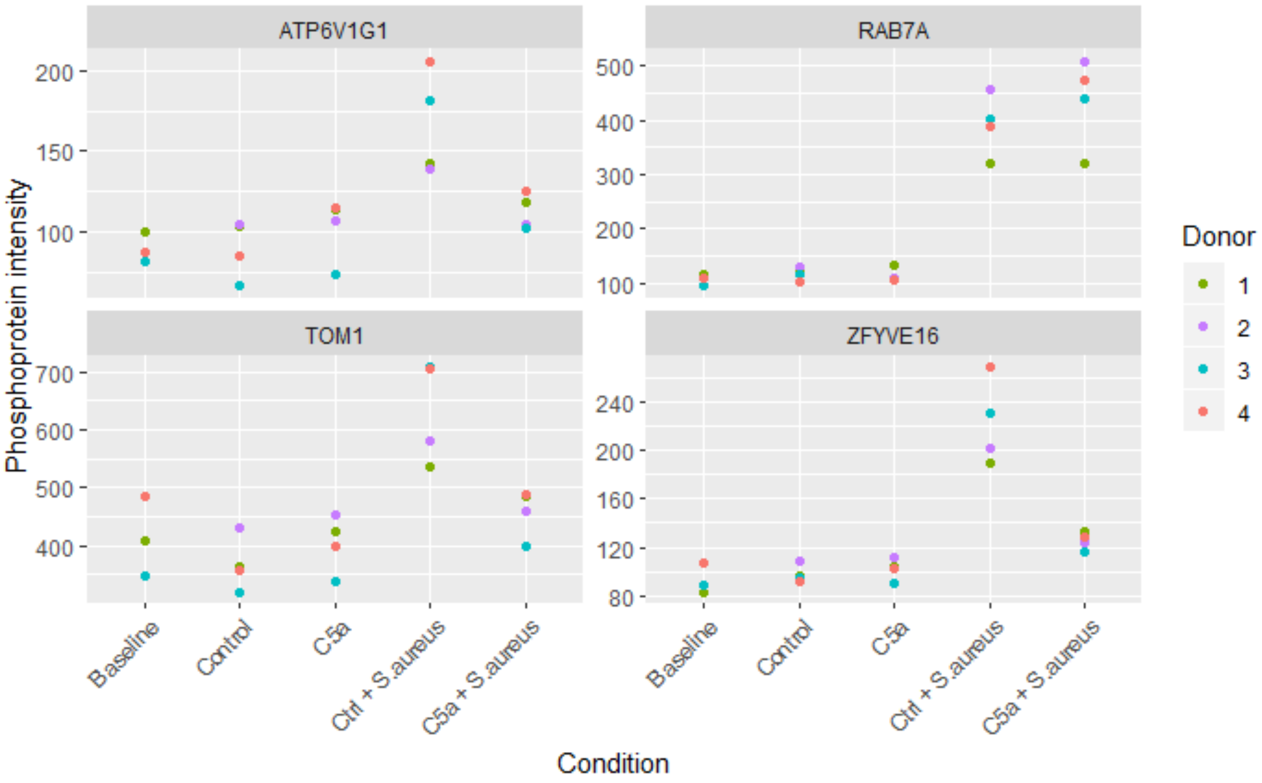


**Supplemental figure 6: *S. aureus* and C5a affect signalling of key antibacterial proteins**

Phosphoprotein intensities across all conditions for 4 key proteins (discussed in detail in text) are shown for each individual donor. TOM1, ZFYVE16, ATP6V1G1 and RAB7a are all involved in phagosomal maturation.

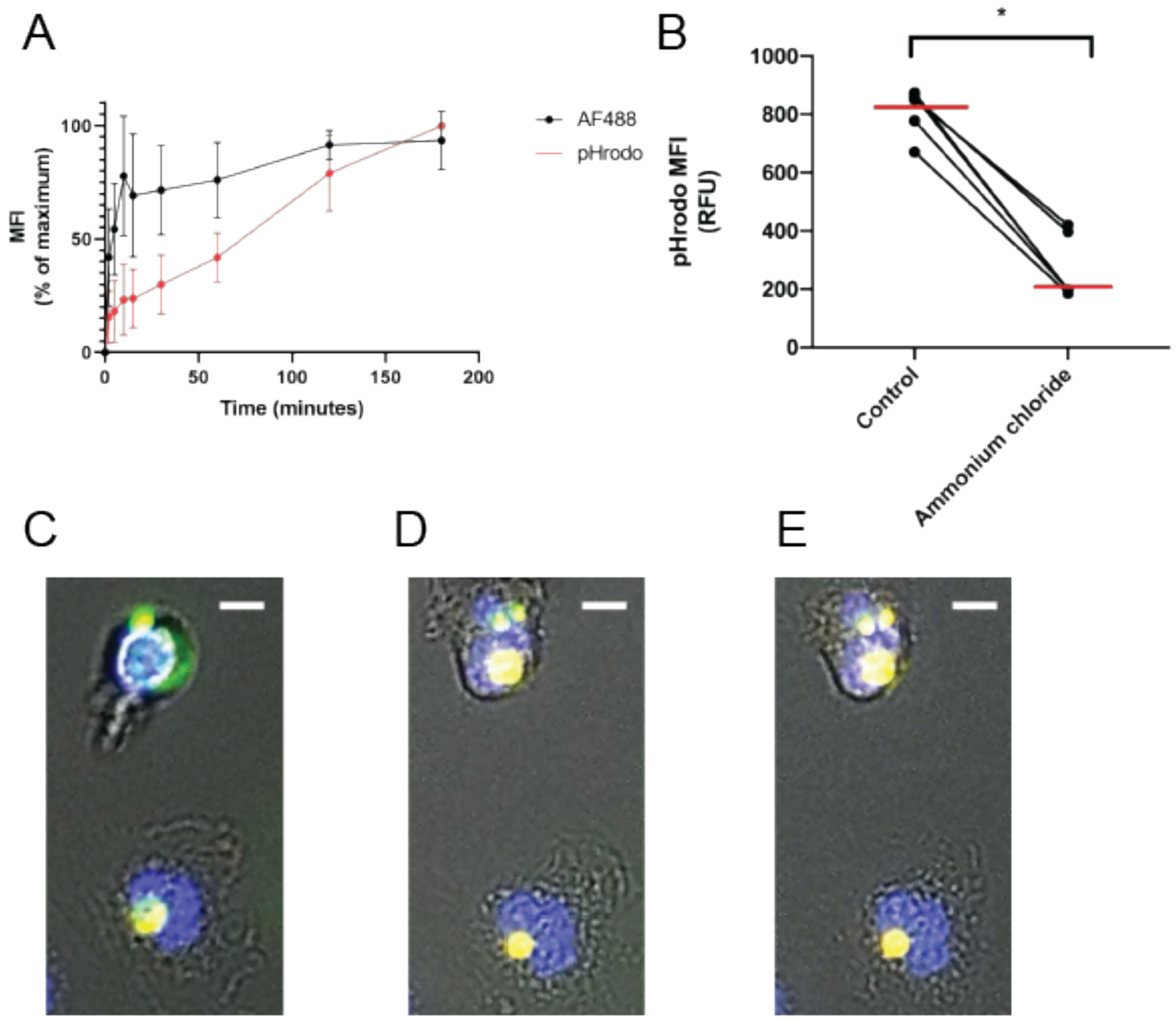


**Supplemental figure 7: internalisation and acidification are distinct processes measured by combined AF488/pHrodo™ probe**

**A:** Whole blood was incubated with dual-labelled phagocytosis probe (10 µg/mL) for 200 minutes as detailed in the Methods section of the main manuscript. Phagocytosis is indicated by AF488 signal and phagosomal acidification by pHrodo™ signal indicating different kinetics and readouts for these two processes. Data are shown as mean ± SD of n = 4 individual donors. $p<0.0001$ by two way ANOVA, $p=0.012$ for difference between AF488 and pHrodo, $p<0.0001$ for time.

**B:** Whole blood was incubated with pHrodo™-labelled *S. aureus* bioparticles (5 µg/mL) for 120 minutes prior to addition of 15mM ammonium chloride or control for 5 minutes before

measurement of pHrodo MFI by flow cytometry as indicated in methods. Data with medians for n = 6 individual donors. * $p < 0.05$ by Wilcoxon.

**C-E:** Purified neutrophils were imaged during phagocytosis using an ArrayScan XTI high throughput screening microscope (20X objective) as detailed in supplemental methods. Stills of neutrophils phagocytosing dual-labelled AF488 (green)/pHrodo™(red) probe at 5 (C) 10 (D) and 25 (E) minutes cropped from Video S1, demonstrating increasing pHrodo signal with resultant colour change from green to yellow (green + red) with time in the absence of further particle ingestion. Scale bar = 5 μM

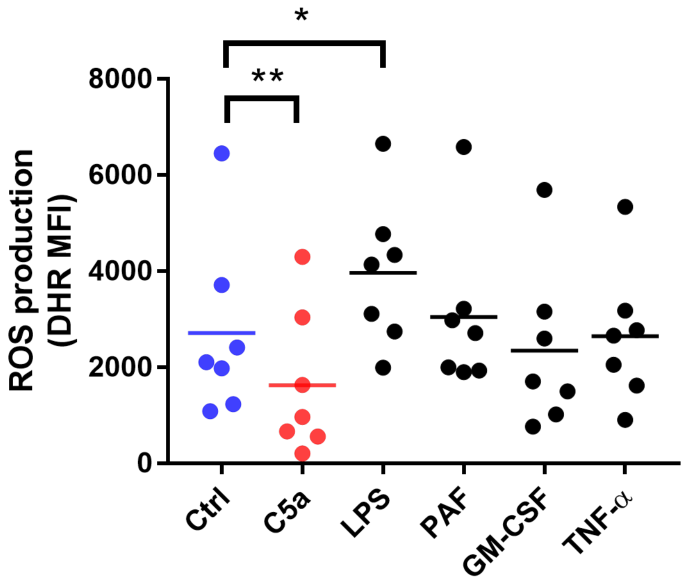


**Supplemental figure 8: C5a pre-treatment reduces ROS production associated with *S. aureus* phagocytosis**

Whole blood was exposed to a combined pHrodo and DHR-labelled probe for 30 minutes after pre-treatment with various agonists. Concentrations and pre-treatment durations were as follows: Ctrl (PBS) 60 minutes; C5a 300 nM, 60 minutes; LPS 100 ng/mL, 60 minutes; PAF 1 μM, 5 minutes; GM-CSF 10 ng/mL, 30 minutes; TNF-α 20 ng/mL, 30 minutes. ANOVA p-value $< 0.0001$ for both D and E, *$p < 0.05$, **$p < 0.01$ for Dunnett's multiple comparisons. Data are shown as individual data points with means for n = 7 individual donors.

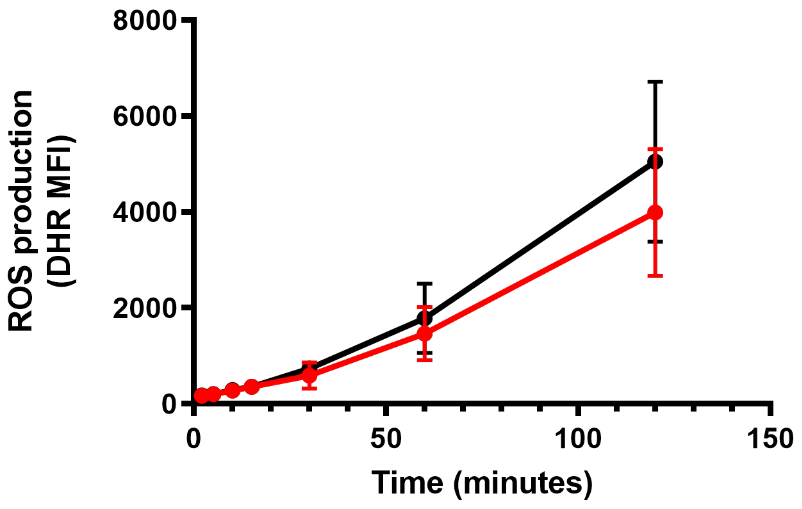


**Supplemental figure 9: VPS34-IN1 did not significantly reduce phagosomal ROS production.**

Whole blood was pre-treated with 1 µM VPS34-IN1 nM for 60 minutes prior to addition of the phagosomal maturation probe. Cells were allowed to phagocytose for the time points indicated. ROS production was quantified by flow cytometry. Data are shown as mean +/- SD of n = 5 independent experiments. No significant difference by repeated measures two-way ANOVA with Bonferroni's multiple comparisons test.

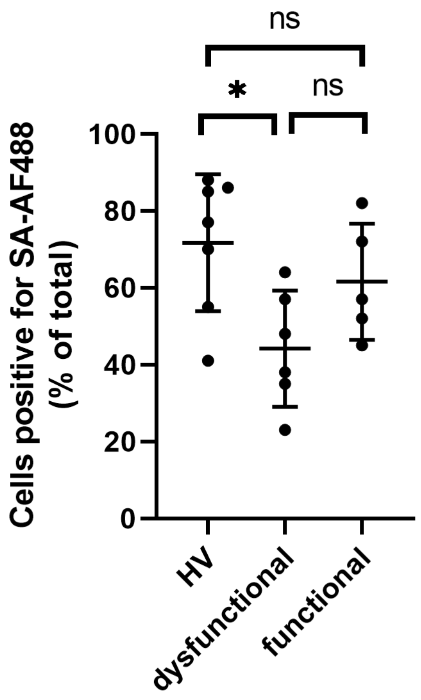


**Supplemental figure 10: reduced *S. aureus* dual labelled probe phagocytosis in patients with dysfunctional neutrophils**

Critically ill patients were identified as having functional or dysfunctional neutrophils using an assay of zymosan phagocytosis previously validated in critically ill patients (12-14, 46). Whole blood was incubated with 5 µg/mL maturation probe for 120 minutes before cells were stained, RBCs lysed and analysed by flow cytometry as detailed in Methods. Data are shown as mean ± SD for 7 healthy volunteers (HV) and 11 patients (6 dysfunctional, 5 functional). *$p < 0.05$ by Kruskal-Wallis.

# Supplemental methods

**Reagents**

Reagents included purified human C5a (ComplementTech, Tyler, TX, USA), Argatroban (R&D Systems, Abingdon UK), recombinant human PAF (Tocris Bioscience, Abingdon, UK), recombinant human TNFa (R&D Systems, Abingdon, UK), recombinant human GM-CSF and *E. coli*-derived LPS (ThermoFisher Scientific, Waltham, MA, USA) and VPS34IN1 (Selleckchem, Houston, TX, USA). pHrodo™ and AF488-conjugated *E. coli* and *S. aureus* bioparticles were from ThermoFisher Scientific (Waltham, MA, USA) and DHR was from CalBiochem (Watford, UK). Antibodies used were: FITC-conjugated mouse anti-human CD88/C5aR1, clone S5/1 (BioRad, Hertfordshire, UK) and Pacific Blue-mouse anti-human CD16, clone 3G8 (Biolegend, London, UK).

**No-wash, no-lyse whole blood assay of neutrophil phagocytosis and ROS production**

This assay was adapted from a protocol provided by Life Technologies designed for use with the Attune Nxt™ acoustic focussing cytometer, see link:
https://www.thermofisher.com/content/dam/LifeTech/global/life-sciences/cellanalysis/Files/0415/Attune-No-WashLyseDetectionLeukocytes-App-Note.pdf
The initial choice of anticoagulant was informed by work from Mollnes and colleagues (Mollnes et al, 2002) which showed that direct thrombin inhibitors allowed complement activation and minimally affected leukocyte function. Blood was treated with inhibitors or priming agents as indicated in respective figure legends. After treatment, 50 µL of blood was aliquoted into 96 well plates in triplicate and a combined *S. aureus* pHrodo™/DHR probe was added at final concentrations of 15 µg/mL and 3 µM respectively. Volume was made up to 100 uL with RPMI 1640 and plates were incubated at 37 °C and 5 % $CO_2$ for 30 minutes to allow phagocytosis to occur. 5 uL from each well was transferred into ice-cold RPMI 1640 media in flow cytometry tubes and stained with anti-CD16 antibody at 4C for 30 minutes.

Volume was made up to 4 mL with ice-cold PBS before the cells were analysed on an Attune Nxt™ Acoustic Focusing Cytometer (LifeTechnologies, Paisely, UK). Phagocytosis was quantified by the percentage of positive neutrophils (with a sample incubated at 4°C serving as a negative control), and median fluorescent intensity of the positive cells, which were multiplied together to give a phagocytic index. In the dual target exposure two pHrodo *S. aureus* Bioparticles with different fluorescent markers were used; pHrodo green and pHrodo red were used. 50 µL of blood was aliquoted into 96 well plates in triplicate and the first Bioparticle *S. aureus* pHrodo red was added at a final concentration indicated in the figure legend and incubated for 30 minutes at 37 °C and 5 % $CO_2$ to allow phagocytosis to occur. The second Bioparticle *S. aureus* pHrodo green was then added and cells were allowed to phagocytose as before. Samples were then stained for CD16 and analysed as above.

**Bacterial killing assay – whole blood**

Methicillin-sensitive *Staphylococcus aureus* (MSSA) bacteria (strain ASASM6, kind gift from Prof Gordon Dougan, University of Cambridge) were sub-cultured in 10 mLs of Luria broth (LB) overnight, then grown to early log-phase. Human blood was collected into argatroban (300 µg/mL) and aliquoted into Eppendorf tubes (50 µL). 10 µL of bacterial suspension was added and blood was incubated on a shaker at 200 rpm, at 37˚C for 1 hour. Human cells were lysed by addition of 450 µL pH 11 distilled water for 3 minutes. Samples were vortexed for 15 seconds and lysates diluted 1:1000 with LB then 100 µL of diluted sample was plated on blood agar plates. Plates were incubated overnight at 37˚C and colonies were counted by visual inspection the next morning.

**Bacterial killing assay – purified neutrophils**

Methicillin-sensitive *Staphylococcus aureus* (MSSA) bacteria (strain ASASM6) were sub-cultured in 10 mLs of Luria broth (LB) overnight, then grown to early log-phase. In the

interim, isolated neutrophils were prepared at a concentration of 5 million/mL in IMDM before pre-incubation with 100nM C5a or vehicle control for 60 minutes before exposure to MSSA at an. average multiplicity of infection (MOI) of 10:1, for one hour at 200 rpm, 37 °C. Samples were then treated with distilled water for 3 minutes to lyse the neutrophils without adversely affecting MSSA viability and serial dilutions plated on blood agar for overnight culture and visual counting of colonies.

### Live cell imaging of purified human neutrophils

Human neutrophils were purified from whole blood as detailed in Methods. Neutrophils were resuspended in Live Cell Imaging Solution (Molecular Probes A14291DJ) at a concentration of $10^6$/mL with 10 % autologous serum and Hoescht 33342 (ThermoFisher H3570) diluted 1:2000. Neutrophils were plated onto poly-L-lysine-coated (Sigma P4707) 96-well imaging plates (Greiner Bio-One 655096) and allowed to adhere for 30 minutes at 37ºC and 5 % $CO_2$. Autologous serum-opsonized dual-labelled AF488/pHrodo™ probe was added at a concentration of 5µg/mL and plates were incubated at 37ºC and 5 % $CO_2$ in an ArrayScan XTI high throughput screening microscope (ThermoFisher). Images were taken at 1 minute intervals for 60 minutes.

## Proteomics

### Cell lysis and trypsin digestion

Cells were lysed in freshly prepared ice-cold lysis buffer (0.5 % SDS, 0.1 M TEAB containing 1 X HALT protease and phosphatase inhibitors). Cell suspensions were incubated at 90 °C for 5 minutes and sonicated twice for 20 seconds (EpiShear™ Probe Sonicator, Active Motif). The insoluble fraction was removed by centrifugation at 20 000 g for 10 minutes at 4 °C. Lysate protein concentration was determined by Bradford Protein Assay (Bio-Rad, Quick Start).

100 µg of protein per sample was reduced in 5 mM Tris(2-carboxyethyl)phosphine (TCEP) for one hour at 60 °C, which was followed by alkylation of cysteines with 10 mM methyl methanethiosulfonate in the dark at room temperature. Lysates were then diluted 1:10 with 0.1 M TEAB before trypsin was added at a 1:30 trypsin:protein ratio by mass. Digestion was carried out overnight at room temperature.

**Tandem mass tag labelling**

Tandem mass tag (TMT) reagents (0.8 mg, ThermoFisher Scientific) were reconstituted in 40 µl anhydrous acetonitrile and peptide samples were labelled according to the manufacturer's protocol. Following incubation at room temperature for one hour, the reaction was quenched with hydroxylamine to a final concentration of 5% (v/v) for 15 min at room temperature. The TMT-labelled samples were pooled at 1: 1: 1: 1: 1: 1: 1: 1: 1: 1: 1 across the 11 samples. The pooled sample was vacuum centrifuged to dryness and subjected to basic pH reversed-phase (bRP) fractionation.

**Off-line reverse phase fractionation at basic pH**

The TMT mixture was fractionated on a Dionex Ultimate 3000 system (Thermo Fisher Scientific) at high pH. The labelled peptide mixture was reconstituted in 20 mM ammonium hydroxide in water (pH 10) and subjected to a 45 minute linear gradient from 4.5% to 45% acetonitrile in 20 mM ammonium hydroxide (pH 10) at a flow rate of 0.2 ml/min over an XBridge C18 column Reversed-Phase (3.5 µm particles, 2.1 mm ID, 150 mm in length; Waters). The peptide mixture was fractionated into a total of 48 fractions. 15% (v/v) of each fraction was separated into a new tube and was used for full proteome analysis. The remaining volume in each fraction will be subjected to phosphopeptide enrichment. All fractions were dried on a centrifugal vacuum concentrator.

**Phosphopeptide enrichment**

The initial 48 fractions were consolidated into 14 and submitted to phosphopeptide enrichment by using the High-Select™ Fe-NTA Phosphopeptide Enrichment Kit according to the manufacturer's protocol. Eluates containing the phosphopeptides were dried via vacuum centrifugation and reconstituted in 10 µl of 0.1 % formic acid for liquid chromatography and tandem mass spectrometry (LC-MS/MS) processing.

**LC-MS/MS analysis of full proteome fractions**

The initial 48 fractions were pooled into a final number of 32 fractions. Each fraction was reconstituted in 10 µl of 0.1% formic acid and 5 µl were analysed on a Dionex Ultimate 3000 UHPLC system coupled with an Orbitrap Fusion Lumos mass spectrometer (Thermo Fisher Scientific, San Jose, CA). Samples were loaded on an Acclaim PepMap 100, 100 µm × 2 cm C18, 5 µm, 100 Å trapping column with the ulPickUp injection method at a loading flow rate of 5 µL/min for 10 min. For peptide separation, an EASY-Spray analytical column 75 µm × 25 cm, C18, 2 µm, 100 Å column was used for multi-step gradient elution at a flow rate of 300 nL/min. Mobile phase (A) was composed of 2 % acetonitrile, 0.1 % formic acid; mobile phase (B) was composed of 80 % acetonitrile, 0.1 % formic acid. Peptides were eluted using a gradient as follows: 0 - 10 min, 5 % mobile phase B; 10 – 95 min, 5 – 45% mobile phase B; 95 -100 min, 45% - 95% B; 100 - 108 min, 95% B; 108 – 110min, 95% - 5% B; 110 – 120 min, 5% B.

Data-dependent acquisition began with an MS survey scan in the Orbitrap (380 – 1500 m/z, resolution 120,000 full width half maximum (FWHM), automatic gain control (AGC) target 3E5, maximum injection time 100 ms). The top ten precursors were then selected for MS2/MS3 analysis. MS2 analysis consisted of: collision-induced dissociation (CID), quadrupole ion trap analysis, AGC target 1E4, normalized collision energy (NCE) 35, q-value 0.25, maximum injection time 35 ms, an isolation window at 0.7, and a dynamic

exclusion duration of 45 seconds. Following acquisition of MS2 spectrum, MS3 precursors were fragmented by high energy collision-induced dissociation (HCD) using 10 frequency notches and analysed in the Orbitrap (resolution 50,000 FWHM, AGC target 5E4, NCE 55, maximum injection time 86 ms, and isolation window at 0.7.

**LC-MS/MS analysis of phosphopeptide-enriched fractions**

All the fractions (10 µl) were analysed on a Dionex Ultimate 3000 UHPLC system coupled to a Q-Exactive HF mass spectrometer (Thermo Fisher Scientific, San Jose, CA). Samples were loaded on an Acclaim PepMap 100, 100 µm × 2 cm C18, 5 µm, 100 Å trapping column with the ulPickUp injection method at a loading flow rate of 5 µL/min for 10 min. For peptide separation, an EASY-Spray analytical column 75 µm × 25 cm, C18, 2 µm, 100 Å column was used for multi-step gradient elution at a flow rate of 300 nL/min. Mobile phase (A) was composed of 2 % acetonitrile, 0.1 % formic acid, 5 % dimethyl sulfoxide (DMSO); mobile phase (B) was composed of 80 % acetonitrile, 0.1 % formic acid, 5 % DMSO. Peptides were eluted using a gradient as follows: 0 - 10 min, 5 % mobile phase B; 10 – 95 min, 5 – 45 % mobile phase B; 95 -100 min, 45 % - 95% B; 100 - 108 min, 95 % B; 108 – 110min, 95 % - 5 % B; 110 – 120 min, 5 % B.

Data-dependent acquisition began with an MS survey scan in the Orbitrap (400 – 1600 m/z, resolution 60,000 FWHM, AGC target 3E6, maximum injection time 100 ms). The top 10 precursors were then isolated and fragmented with CID. MS2 analysis consisted of: resolution 30,000 FWHM, AGC target 2E4, NCE 33, maximum injection time.

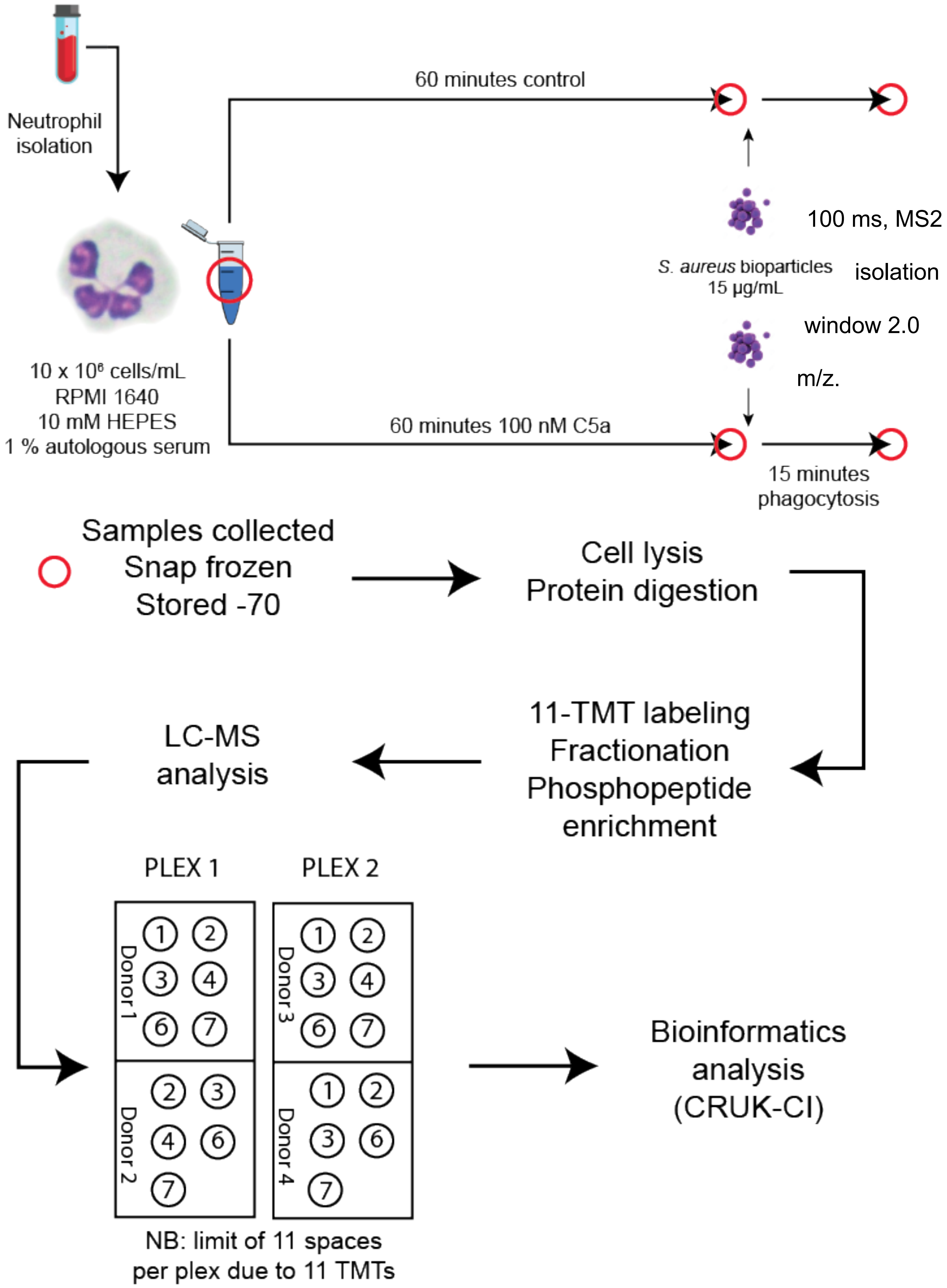

Neutrophil isolation
10 x 10⁶ cells/mL
RPMI 1640
10 mM HEPES
1 % autologous serum
60 minutes control
60 minutes 100 nM C5a
S. aureus bioparticles
15 µg/mL
100 ms, MS2
isolation
window 2.0
m/z.
15 minutes
phagocytosis
Samples collected
Snap frozen
Stored -70
Cell lysis
Protein digestion
11-TMT labeling
Fractionation
Phosphopeptide
enrichment
LC-MS
analysis
PLEX 1
PLEX 2
Donor 1
Donor 2
Donor 3
Donor 4
1
2
3
4
6
7
NB: limit of 11 spaces
per plex due to 11 TMTs
Bioinformatics
analysis
(CRUK-CI)

**Supplemental figure 11: Schematic of phosphoproteomic workflow**

Neutrophils were purified as described and suspended in RPMI 1640 media supplemented with 10 mM HEPES and 1% AS. Red circles indicate instances where aliquots of cells were removed for analysis. An untreated aliquot was removed and snap frozen and cells were then treated with 100 nM C5a, or control for 60 minutes. Post-treatment, pre-phagocytosis aliquots were removed and snap frozen, and pHrodo™ *S. aureus* Bioparticles were added; cells were allowed to phagocytose for 15 minutes before the final aliquots were removed and snap frozen. Phagocytosis was measured at the end of the procedure by flow cytometry of remaining (non-frozen) sample. Samples were batched until all 22 conditions were ready for analysis. Samples were transported to CRUK-CI on dry ice, lysed and digested as specified. The phosphoproteomic workflow included TMT labelling, fractionation and enrichment of phosphopeptides prior to LC-MS analysis in two separate batches or plexes with the 22 samples from four donors distributed across them as shown.
